# Elesclomol Loaded Copper Oxide Nanoplatform Triggers Cuproptosis to Enhance Antitumor Immunotherapy

**DOI:** 10.1002/advs.202309984

**Published:** 2024-03-02

**Authors:** Xufeng Lu, Xiaodong Chen, Chengyin Lin, Yongdong Yi, Shengsheng Zhao, Bingzi Zhu, Wenhai Deng, Xiang Wang, Zuoliang Xie, Shangrui Rao, Zhonglin Ni, Tao You, Liyi Li, Yingpeng Huang, Xiangyang Xue, Yaojun Yu, Weijian Sun, Xian Shen

**Affiliations:** ^1^ Department of Gastrointestinal Surgery Zhejiang International Scientific and Technological Cooperation Base of Translational Cancer Research The Second Affiliated Hospital and Yuying Children's Hospital of Wenzhou Medical University Wenzhou 325000 China; ^2^ Research Center of Basic Medicine The Second Affiliated Hospital and Yuying Children's Hospital of Wenzhou Medical University Wenzhou Zhejiang 325000 China; ^3^ Department of Gastrointestinal Surgery The First Affiliated Hospital of Wenzhou Medical University Wenzhou Zhejiang 325000 China; ^4^ Wenzhou Collaborative Innovation Center of Gastrointestinal Cancer in Basic Research and Precision Medicine Wenzhou Key Laboratory of Cancer‐related Pathogens and Immunity Department of Microbiology and Immunology Institute of Molecular Virology and Immunology Institute of Tropical Medicine School of Basic Medical Sciences Wenzhou Medical University Wenzhou 325000 China; ^5^ Key Laboratory of Laboratory Medicine Ministry of Education School of Laboratory Medicine and Life Sciences Wenzhou Medical University Wenzhou Zhejiang 325000 China

**Keywords:** CuO, cuproptosis, elesclomol, immunotherapy, PD‐1

## Abstract

The induction of cuproptosis, a recently identified form of copper‐dependent immunogenic cell death, is a promising approach for antitumor therapy. However, sufficient accumulation of intracellular copper ions (Cu^2+^) in tumor cells is essential for inducing cuproptosis. Herein, an intelligent cuproptosis‐inducing nanosystem is constructed by encapsulating copper oxide (CuO) nanoparticles with the copper ionophore elesclomol (ES). After uptake by tumor cells, ES@CuO is degraded to release Cu^2+^ and ES to synergistically trigger cuproptosis, thereby significantly inhibiting the tumor growth of murine B16 melanoma cells. Moreover, ES@CuO further promoted cuproptosis‐mediated immune responses and reprogrammed the immunosuppressive tumor microenvironment by increasing the number of tumor‐infiltrating lymphocytes and secreted inflammatory cytokines. Additionally, combining ES@CuO with programmed cell death‐1 (PD‐1) immunotherapy substantially increased the antitumor efficacy in murine melanoma. Overall, the findings of this study can lead to the use of a novel strategy for cuproptosis‐mediated antitumor therapy, which may enhance the efficacy of immune checkpoint inhibitor therapy.

## Graphical abstract

A copper oxide (CuO)‐based and copper ionophore elesclomol (ES)‐loaded nanoplatform (denoted ES@CuO) is designed to trigger immunogenic cell death via cuproptosis. Meanwhile, combined therapy with ES@CuO nanoparticles and PD‐1 synergistically remodels the immunosuppressive tumor microenvironment to significantly inhibit the growth of murine melanoma.

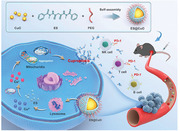

## 1 Introduction

Melanoma is the most lethal type of skin cancer and originates from the malignant transformation of melanocytes.^[^ ^1^, ^2^ ^]^ Melanoma is the fifth most common cancer worldwide and the leading cause of skin cancer‐related death.^[^ ^3^, ^4^ ^]^ This disease frequently metastasizes to and can spread to distant organs such as the brain, lung, liver, and bone through lymph nodes and blood vessels.^[^ ^5^, ^6^ ^]^ Currently, immune checkpoint inhibitors (ICIs), represented by programmed cell death‐1 (PD‐1) antibodies, have shown outstanding therapeutic efficacy for patients with immunogenic melanoma in the clinic.^[^ ^7^, ^8^ ^]^ Despite its ability to substantially potentiate therapeutic efficacy, current PD‐1‐based immunotherapies are limited by a low response rate owing to the immunosuppressive tumor microenvironment (TME) and suppressed antitumor immunity.^[^ ^7^, ^8^ ^]^ With the continuously increasing incidence of melanoma, enhancing the efficacy of PD‐1 therapy and developing alternative therapeutic approaches are urgently needed to improve the survival of patients with melanoma.

The induction of cuproptosis, a novel form of regulated cell death, has emerged as a promising strategy for tumor therapy.^[^ ^9^, ^10^, ^11^, ^12^, ^13^, ^14^, ^15^, ^16^, ^17^ ^]^ Cuproptosis is triggered by the accumulation of intracellular copper ions (Cu^2+^), which subsequently bind to lipoylated components in the mitochondrial tricarboxylic acid (TCA) cycle, aggregating Cu‐bound lipoylated mitochondrial proteins and downregulating Fe‐S (iron‐sulfur) cluster proteins, resulting in proteotoxic stress and ultimately programmed cell death.^[^ ^18^, ^19^ ^]^ Cuproptosis is a form of Cu‐dependent immunogenic cell death (ICD) involving immune responses through the release of many damage‐associated molecular patterns (DAMPs) and tumor‐associated antigens.^[^ ^20^, ^21^ ^]^ Therefore, cuproptosis could reverse the immunosuppressive TME and subsequently enhance the efficacy of ICI‐based immunotherapy. However, cuproptosis is strongly dependent on the continuous accumulation of Cu^2+^ in mitochondria, and intracellular Cu^2+^ in tumor cells is strongly restricted by glutathione, Cu^2+^‐transporting proteins, and metabolic proteins, which are major obstacles to effectively inducing cuproptosis.^[^ ^22^, ^23^ ^]^ Therefore, further enhancing the accumulation of intracellular Cu^2+^ in tumor cells is highly desirable for cuproptosis‐based antitumor therapy.

**Scheme 1:**
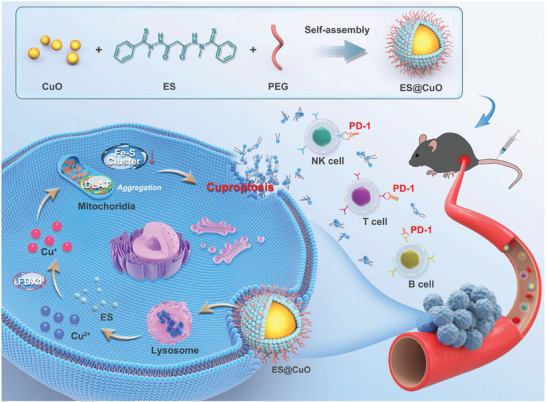
Schematic illustration of the mechanism of a cuproptosis‐inducing nanosystem (denoted ES@CuO) combined with PD‐1 for synergistic tumor immunotherapy in melanoma.

As metal oxide nanoparticles with excellent characteristics, copper oxide nanoparticles (CuO NPs) have been used as outstanding drug delivery platforms for antitumor and antimicrobial therapy because of their considerable toxicity, high drug loading potential, and biocompatibility.^[^ ^24^, ^25^ ^]^ Furthermore, CuO NPs successfully enter cells by endocytosis and liberate Cu^2+^ in the acidic environment of lysosomes, eventually leading to cellular toxicity.^[^ ^26^ ^]^ In this regard, CuO NPs are candidates for copper‐based nanocarriers that are likely to efficiently trigger cuproptosis. Therefore, the copper ionophore elesclomol (ES) and CuO NPs were encapsulated in polyethylene glycol polymer (PEG) to construct a cuproptosis‐inducing nanosystem (denoted ES@CuO). After uptake by tumor cells, ES@CuO could be degraded to release Cu^2+^ and ES simultaneously in an acidic environment. Subsequently, the liberated Cu^2+^ and ES directly targeted transportation to mitochondria and then caused dihydrolipoyl transacetylase (DLAT) aggregation, resulting in cuproptosis in B16 tumor cells. Notably, cuproptosis induces damage to the cell membrane to release many DAMPs and effectively induces an immune response, which further promotes abundant lymphocyte infiltration and the secretion of inflammatory cytokines and subsequently reprograms the immunosuppressive TME in tumor tissues, thereby inhibiting tumor growth in B16 tumor‐bearing mice. Moreover, combining ES@CuO nanoparticles with PD‐1 therapy favorably improved the antitumor efficacy of ICI immunotherapy. Thus, ES@CuO might be a promising nanomedicine for melanoma treatment by inducing cuproptosis‐based immunotherapy (**Scheme** **1**).

## 2 Results and Discussion

### 2.1 Preparation and Characterization of ES@CuO

**Figure 1:**
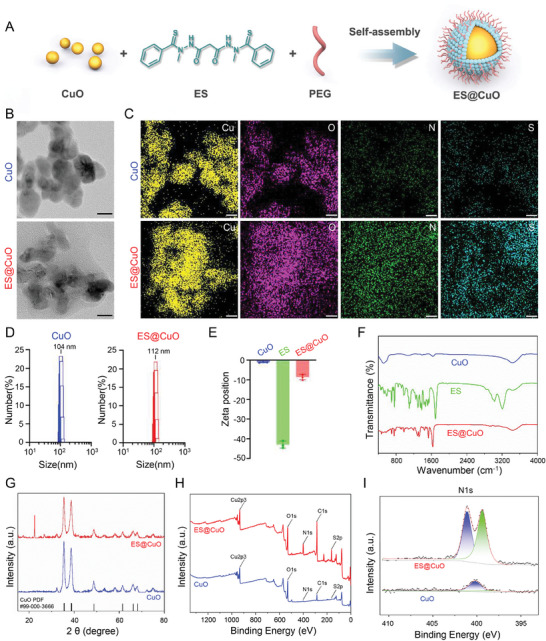
Preparation and characterization of ES@CuO. A) Schematic diagram of the preparation of ES@CuO NPs. B) Representative transmission electron microscopy (TEM) images of CuO NPs and ES@CuO NPs. Scale bar: 20 nm. C) Representative element maps (Cu, O, N, and S) of CuO NPs and ES@CuO NPs. Scale bar: 20 nm. ES formula: C_19_H_20_N_4_O_2_S_2_. D) and E) The hydrodynamic size distributions and zeta potentials of CuO NPs and ES@CuO NPs (in ultrapure water) (*n* = 3). F) Fourier transform infrared (FTIR) spectra of CuO NPs, ES, and ES@CuO NPs. G) XRD analysis of CuO NPs and ES@CuO NPs. H) XPS analysis of CuO NPs and ES@CuO NPs. I) High‐resolution N1s XPS spectra of CuO NPs and ES@CuO NPs. The data are presented as the mean ± SD.

Briefly, the ES@CuO nanoplatform was fabricated as shown in **Figure** **1**A. The physicochemical characteristics of the ES@CuO NPs were subsequently investigated. Transmission electron microscopy (TEM) images showed the homogeneous spherical structure of ES@CuO and its surface with an ≈8 nm‐thick membrane structure, indicating successful surface coating (Figure 1B). Elemental mapping of the composition of the ES@CuO NPs with Cu, O, N, and S confirmed the successful encapsulation of ES on the CuO NPs (Figure 1C). According to the dynamic light scattering measurement results, the particle sizes of CuO and ES@CuO were 104 ± 11 and 112 ± 16 nm in diameter, respectively (Figure 1D). Moreover, the zeta potentials of CuO and ES in ultrapure water were ≈−1 and −49 mV, respectively (Figure 1E). After ES adsorption, the zeta potential of CuO decreased from −1 to –10 mV, indicating electrostatic attraction between the CuO NPs and ES, which may be attributed to the chelation of Cu^2+^‐ES (Figure 1E). ES@CuO was further characterized by ultraviolet‒visible (UV‒vis) and Fourier transform infrared (FTIR) analyses. UV‒vis spectrophotometry of ES@CuO revealed an increase in absorption at 390 nm, which was the characteristic absorption peak of ES, demonstrating the successful loading of ES (Figure S1, Supporting Information). As shown by UV‒vis spectrophotometry, the drug loading content (LC%) of the ES was calculated to be ≈95% (Figure S2, Supporting Information). Moreover, the presence of characteristic peaks of ES in the FTIR spectrum of ES@CuO provided further evidence of ES@CuO generation (Figure 1F). Furthermore, the X‐ray diffraction (XRD) patterns verified that the obtained ES@CuO also contained the characteristic peaks of CuO NPs (Figure 1G). In addition, X‐ray photoelectron spectroscopy (XPS) confirmed that ES@CuO consists of Cu, O, N, and S, indicating the successful preparation of ES@CuO (Figure 1H,I; Figure S3, Supporting Information). Collectively, the above results demonstrated the successful fabrication of the ES@CuO NPs.

### 2.2 pH‐Triggered Degradation

Acidic conditions can trigger the degradation of CuO NPs,^[^ ^27^ ^]^ leading to the dissociation of ES@CuO and further release of Cu^2+^ and ES. We explored the pH‐sensitive dissociation of ES@CuO, which induces the release of Cu^2+^ and ES. As expected, inductively coupled plasma‒mass spectrometry (ICP‒MS) results showed that ≈3.1% of the Cu^2+^ was released from the ES@CuO NPs after 24 h of incubation at pH 4.5, while ≈1.3% of the Cu^2+^ was released from the ES@CuO NPs at pH 5.5. In contrast, ES@CuO in neutral phosphate‐buffered saline (PBS) simultaneously released ≈0.5% of the material from ES@CuO (Figure S4A–C, Supporting Information). We further investigated the synchronous release of ES from ES@CuO due to the destruction of the PEG membrane structure. As shown in Figure S4D (Supporting Information), the corelease of ES detected by high‐performance liquid chromatography (HPLC) showed a similar pH‐dependent pattern, further demonstrating the successful formation of ES@CuO. These results indicated that ES@CuO, which has pH‐sensitive drug release behavior, could be slowly degraded to release Cu^2+^ and ES in an acidic microenvironment.

### 2.3 Cellular Uptake

**Figure 2:**
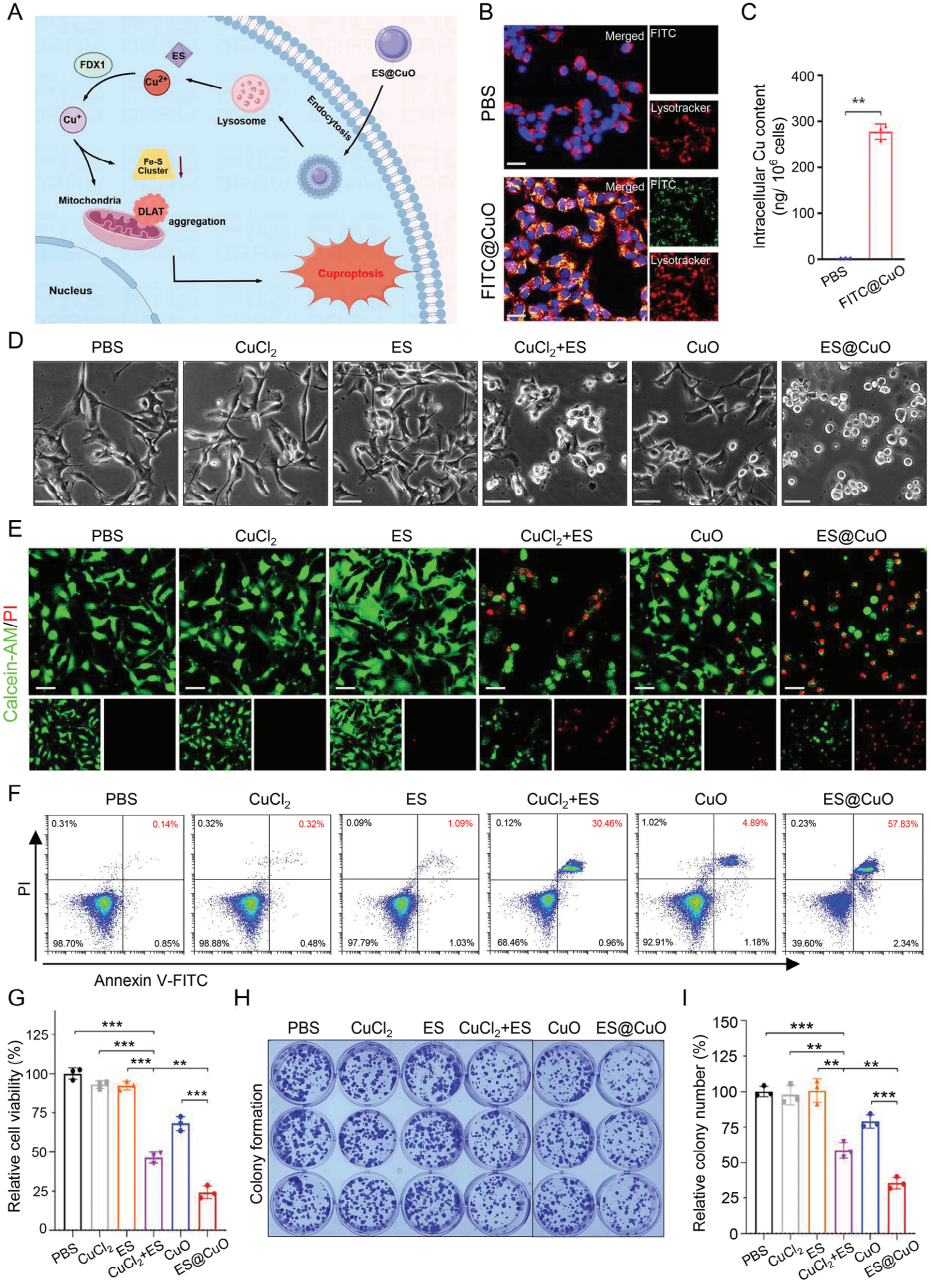
In vitro antitumor effect of ES@CuO on B16 tumor cells. A) Schematic of the possible mechanism of ES@CuO‐induced cuproptosis. B) Representative confocal microscopy images of B16 cells after incubation with FITC@CuO NPs for 6 h. The cell nuclei were stained with Hoechst 33342 (blue), and the lysosomes were stained with LysoTracker (red). Scale bars: 25 µm. C) Intracellular uptake of Cu by B16 cells after incubation with FITC@CuO NPs for 6 h. D) Representative morphological changes in B16 cells after different treatments. Scale bar: 50 µm. E) Live/dead staining of B16 cells after different treatments. Scale bar: 50 µm. F) After the indicated treatments, B16 tumor cells were stained with Annexin V‐FITC and PI and then analyzed by flow cytometry. G) Cell viability of B16 cells after 24 h of incubation with the indicated treatments (*n* = 3). H) Colony of B16 cells treated with different NPs and I) the corresponding quantification of colony number (*n* = 3). The data are presented as the mean ± SD; p values were calculated using an unpaired, 2‐tailed Student's t test with Welch's correction; **p < 0.01; ***p < 0.001.

In the past decade, ES has been considered an adjuvant chemotherapy drug and was first applied in clinical trials for melanoma.^[^ ^28^, ^29^ ^]^ Therefore, we chose the murine melanoma cell line B16 as the cell model. To investigate the intracellular uptake of the ES@CuO NPs, we labeled CuO NPs with the fluorescent agent fluorescein isothiocyanate (FITC) to form FITC@CuO NPs (Figure S5, Supporting Information). As shown in Figure S6 (Supporting Information), the fluorescence intensity quantified by flow cytometry was significantly greater in the FITC@CuO‐treated B16 cells than in the other cells. Moreover, confocal laser microscopy (CLSM) analysis of the subcellular localization of the proteins showed that the fluorescence was predominantly located in the lysosomes of the FITC@CuO‐treated B16 cells at 6 h (**Figure** **2**A,B). Subsequently, we determined the copper content in B16 cells using ICP‒MS analysis. The content of intracellular Cu^2+^ (≈277.9 ng 10^−6^ cells) was substantially elevated in B16 cells in the FITC@CuO‐treated group. In contrast, the intracellular Cu^2+^ concentration (≈1.6 ng 10^−6^ cells) in B16 cells in the control group was negative (Figure 2C). The above results indicated that CuO NPs could be ideal copper‐based nanocarriers for efficiently increasing intracellular Cu^2+^ in B16 cells.

### 2.4 Cytotoxicity of ES@CuO In Vitro

Then, we evaluated the cytotoxicity of the ES@CuO NPs on B16 cancer cells in vitro. As shown in Figure 2D, B16 cells incubated with CuCl_2_+ES (positive control) for 4 h exhibited significant disruption of cell membrane integrity. Interestingly, the ES@CuO‐treated B16 cells had the largest number of cells with injured cell membranes. The noticeable morphological changes consistent with copper‐induced cell death were similar to those observed in a previous study.^[^ ^18^ ^]^ The cell death rate of B16 cells following different treatments was determined by calcein‐AM and propidium iodide (PI) staining (Figure 2E). Flow cytometry analysis revealed that the necrosis rate of the B16 cells treated with ES@CuO was 1.9 times greater than that of the cells treated with CuCl_2_+ES (positive control) (Figure 2F; Figure S7, Supporting Information), indicating that ES@CuO could efficiently kill tumor cells in vitro. Subsequently, the in vitro antitumor activity of ES@CuO toward B16 tumor cells was evaluated by a cell counting kit‐8 (CCK‐8) assay. Despite the promising applications of CuO NPs in antitumor therapy, CuO had slight toxic effects on B16 tumor cells, while the ES@CuO group exhibited a significantly enhanced antitumor effect (Figure 2G; Figures S8 and S9, Supporting Information). Furthermore, we verified the antitumor effect of ES@CuO through a plate colony formation assay. Colony formation assays further confirmed that the number of colonies in the ES@CuO group was ≈60% lower than that in the PBS group (Figure 2H,I).

### 2.5 ES@CuO Induced Cuproptosis In Vitro

**Figure 3:**
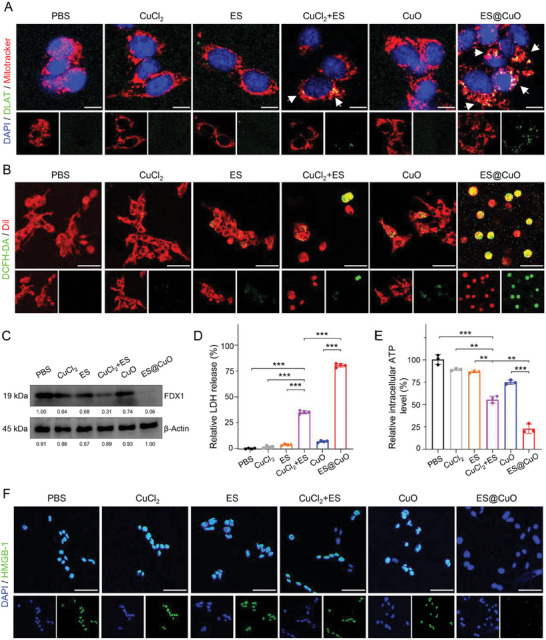
ES@CuO induced cuproptosis in vitro. A) Representative confocal microscopy images of DLAT aggregation in B16 cells after the indicated treatment. The white arrows indicate DLAT aggregation. Scale bars: 10 µm. B) Representative confocal microscopy images of ROS in B16 cells stained with DCFH‐DA fluorescent probes after various treatments. Scale bars: 50 µm. C) Western blot analysis of cuproptosis‐related protein (FDX1) activation in B16 cells after different treatments. D) Lactic dehydrogenase (LDH) release assay of B16 cells following the indicated treatments (n = 4). E) Intracellular ATP levels of B16 cells after 24 h of incubation with the indicated treatments (*n* = 3). F) Representative confocal microscopy images of HMGB‐1 in B16 cells after the indicated treatment. Scale bars: 50 µm. Data are shown as the mean ± SD; p values were calculated using an unpaired, 2‐tailed Student's t test with Welch's correction; **p < 0.01; ***p < 0.001.

During cuproptosis, Cu^2+^ directly binds to DLAT, which induces DLAT aggregation and subsequently results in copper‐mediated cell death.^[^ ^18^, ^30^ ^]^ To investigate the mechanism of ES@CuO‐induced cuproptosis, we visualized DLAT oligomerization via immunofluorescence imaging. As shown in **Figure** **3**A, the cells treated with CuCl_2_, ES, or CuO NPs alone had negligible DLAT foci, while the cells treated with CuCl_2_+ES (positive control) exhibited a greater degree of DLAT aggregation. In particular, pronounced aggregation of DLAT foci was observed in the tumor cells treated with ES@CuO (Figure 3A; Figure S10, Supporting Information). In addition, the Fe‐S cluster protein ferredoxin 1 (FDX1) is a crucial regulator of copper‐triggered cell death and can reduce Cu^2+^ to Cu^+^.^[^ ^18^, ^31^ ^]^ The generated Cu^+^ could react with intracellular H_2_O_2_ to generate toxic ⋅OH via a Fenton‐like reaction (Figure S11, Supporting Information). Therefore, 2,7‐dichlorodihydrofluorescein diacetate (DCFH‐DA) was used to evaluate intracellular reactive oxygen species (ROS) generation after different treatments. As shown in Figure 3B, compared with the almost negligible fluorescence in the control groups, the ES@CuO‐treated group exhibited the most obvious fluorescence, further demonstrating the excellent catalytic performance of the ES@CuO NPs during cuproptosis. Furthermore, FDX1 knockout completely inhibited the accumulation of lipoylated DLAT in mitochondria.^[^ ^18^, ^31^ ^]^ In line with these studies, the knockdown of FDX1 significantly inhibited the ES@CuO‐induced oligomerization of DLAT in B16 cells (Figure S12, Supporting Information). Moreover, we confirmed that the expression of FDX1 was significantly downregulated in the ES@CuO group compared with the other groups (Figure 3C). Overall, our designed ES@CuO NPs could efficiently induce cuproptosis in tumor cells.

### 2.6 ES@CuO‐Triggered Immunogenic Cell Death In Vitro

Recent studies have suggested that cuproptosis can induce ICD to enhance the antitumor immune response.^[^ ^20^, ^21^ ^]^ To evaluate the effect of ICD, three DAMP molecules, high mobility group box‐1 (HMGB‐1), lactate dehydrogenase (LDH), and adenosine triphosphate (ATP), were analyzed. As shown in Figure 3D, the release of cellular LDH significantly increased after treatment with ES@CuO NPs, indicating that ES@CuO‐mediated cuproptosis could dramatically induce destruction of the cell membrane. Consistently, the content of intracellular ATP after ES@CuO treatment was markedly lower than that in the control group, confirming that a large amount of ATP was released into the extracellular environment after ES@CuO treatment (Figure 3E). Furthermore, immunofluorescence images revealed that the fluorescent signals of HMGB‐1 in the nucleus were significantly reduced after ES@CuO treatment, illustrating that ES@CuO promoted the release of nuclear HMGB‐1 (Figure 3F). Overall, these results suggested that ES@CuO effectively enhanced tumor cell immunogenicity by inducing ICD in tumor cells.

### 2.7 In Vivo Biodistribution of ES@CuO

**Figure 4:**
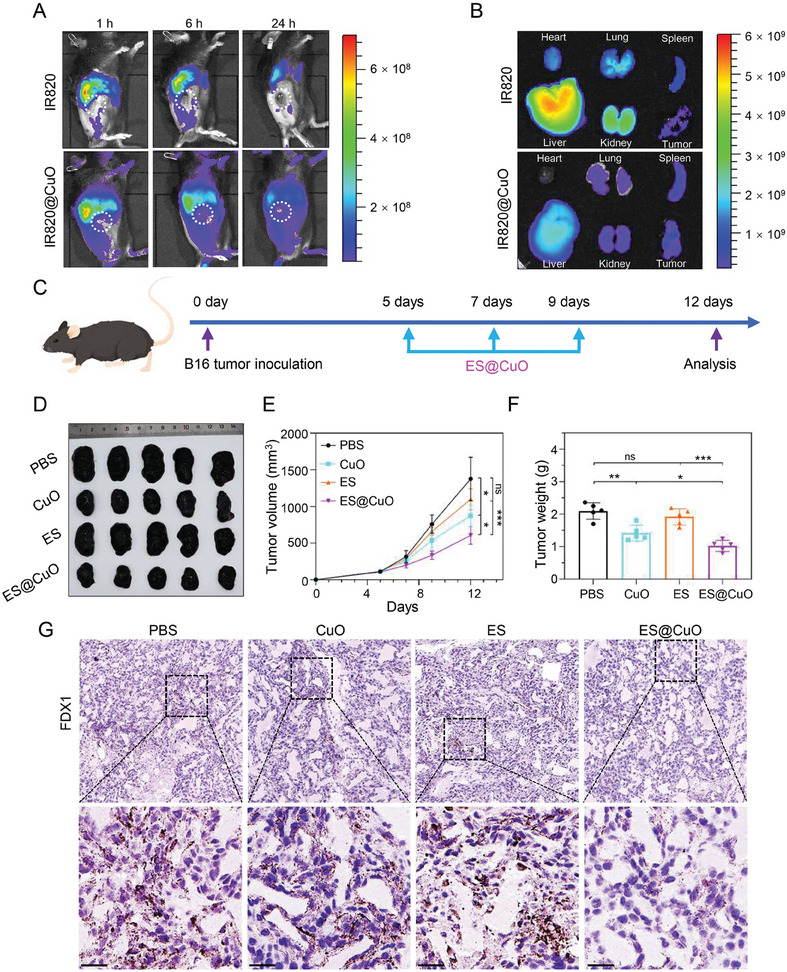
Antitumor effects mediated by ES@CuO in vivo. A) Biodistribution after intravenous injection of free IR820 and IR820@CuO NPs at the indicated time points. B) Ex vivo imaging of IR820 fluorescence intensity in major organs and tumors collected at 6 h post‐injection. C) Schematic illustration of the experimental schedule for B16 tumor‐bearing mice. D) Corresponding tumor photographs, E) tumor growth curves, and F) ex vivo tumor weights of the B16 tumor‐bearing mice after various treatments (*n* = 5). G) IHC analysis of FDX1 expression in tumor tissues after different treatments. Scale bar: 25 µm. Data are shown as the mean ± SD; p values were calculated using an unpaired, 2‐tailed Student's t test with Welch's correction; ns, not significant; *p < 0.05; **p < 0.01; ***p < 0.001.

Given that ES@CuO had significant antitumor activity in vitro, we evaluated the therapeutic effect of ES@CuO in vivo. First, we examined the pharmacokinetic profile of ES@CuO in vivo. B16 tumor‐bearing mice were intravenously injected with new indocyanine green (IR820)‐labeled CuO (IR820@CuO) and monitored by an in vivo optical imaging system at the indicated time points (Figure S13, Supporting Information). The results showed that the fluorescence signal accumulated at the tumor region within 1 h after intravenous injection. In contrast, free IR820 was rapidly cleared from the tumor at 6 h postinjection, whereas there was a continuously strong tumor fluorescence intensity at 24 h after IR820@CuO injection (**Figure** **4**A; Figure S14A, Supporting Information). Subsequently, by determining the ex vivo biodistribution at 6 h postinjection, we found that the ex vivo tumor fluorescence intensity in the IR820@CuO‐injected mice was greater than that in the free IR820‐injected mice, although there was inescapable accumulation in the heart, lung, liver, spleen, and kidney (Figure 4B; Figure S14B, Supporting Information). Overall, these results indicated the long‐term retention and efficient tumor accumulation of the ES@CuO NPs.

### 2.8 Antitumor Ability of ES@CuO In Vivo

Next, we evaluated the potential antitumor effects of ES@CuO in the B16 mouse model. Male C57BJ/6 N mice were subcutaneously injected with B16 tumor cells. When the tumor volume reached ≈80 mm^3^, the mice were randomly divided into four groups (PBS, CuO, ES, and ES@CuO). Mice were intravenously injected with the indicated treatments on days 5, 7, and 9 (Figure 4C). As shown in Figure 4D, the CuO and ES groups displayed limited tumor growth suppression compared with the PBS group. Moreover, the tumor suppression efficacy was greater in the ES@CuO group than in the other groups (Figure 4D–F). Like in vitro, a dramatically decreased expression level of FDX1 was observed in the ES@CuO‐treated tumor tissues, whereas there was a high level of FDX1 in the other groups (Figure 4G; Figure S15, Supporting Information). In addition, we determined the serum levels of HMGB‐1, a major kind of DAMP released by cuproptotic cells.^[^ ^32^ ^]^ Enzyme‐linked immunosorbent assays (ELISAs) confirmed that the ES@CuO‐treated mice released a large amount of HMGB‐1 into the extracellular space (Figure S16, Supporting Information). Taken together, these results indicated that ES@CuO‐induced cuproptosis could inhibit tumor growth in vivo.

### 2.9 ES@CuO NPs Combined with PD‐1 Synergistically Enhanced Antitumor Efficacy

**Figure 5:**
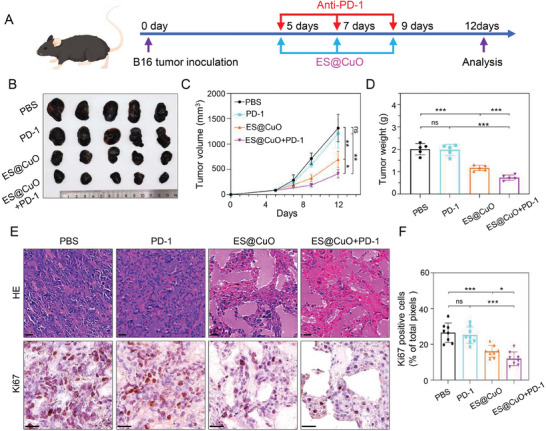
The antitumor efficacy of combined ES@CuO and PD‐1 therapy in vivo. A) Schematic illustration of the therapeutic schedule of combined ES@CuO and PD‐1 therapy in the B16 tumor model. B) Corresponding tumor photographs, C) tumor growth curves, and D) ex vivo tumor weights of the B16 tumor‐bearing mice after the indicated treatments (*n* = 5). E) Representative H&E staining and Ki67 immunohistochemical staining of tumor tissues from mice given various treatments; scale bar: 25 µm. F) Quantification of Ki67 expression in tumor tissue from mice given the indicated treatments (*n* = 8). Data are shown as the mean ± SD; p values were calculated using an unpaired, 2‐tailed Student's t test with Welch's correction; ns, not significant; *p < 0.05; **p < 0.01; ***p < 0.001.

PD‐1, an ICI that enhances T‐cell activity to kill tumor cells, has recently been a popular treatment for antitumor immunotherapy.^[^ ^33^, ^34^ ^]^ Therefore, the above results indicated that treatment with ES@CuO in combination with PD‐1 might be a promising strategy for antitumor therapy. Subcutaneous B16 tumor‐bearing mice were established to further investigate the antitumor efficacy of the combination of ES@CuO and PD‐1. On the 5th, 7th, and 9th days, the mice were administered ES@CuO NPs via intravenous injection and PD‐1 via intraperitoneal injection (**Figure** **5**A). As shown in Figure 5B, the tumor growth of the PD‐1 group was similar to that of the PBS group, further confirming that PD‐1 alone is ineffective at treating an immunosuppressive TME. In comparison, the tumor volume was markedly lower in the mice treated with ES@CuO+PD‐1 than in the mice treated with the other treatments (Figure 5C). Accordingly, the tumor weight data also revealed similar trends (Figure 5D). Subsequently, the therapeutic effect of ES@CuO+PD‐1 was confirmed by hematoxylin and eosin (H&E) staining. Similarly, compared with those in the other groups, the ES@CuO+PD‐1 group displayed the largest area of necrotic cells in the tumor tissues (Figure 5E). In addition, quantitative analysis of Ki67 immunohistochemistry (IHC) staining confirmed the significant tumor inhibitory efficacy of ES@CuO+PD‐1 therapy (Figure 5E,F). Importantly, the combined treatment significantly improved the survival of B16 tumor‐bearing mice compared to that of the other groups (Figure S17, Supporting Information). Taken together, these results suggested that ES@CuO could enhance the antitumor efficacy of PD‐1‐based immunotherapy.

### 2.10 ES@CuO and PD‐1 Combined Immunotherapy Reprogrammed the TME

**Figure 6:**
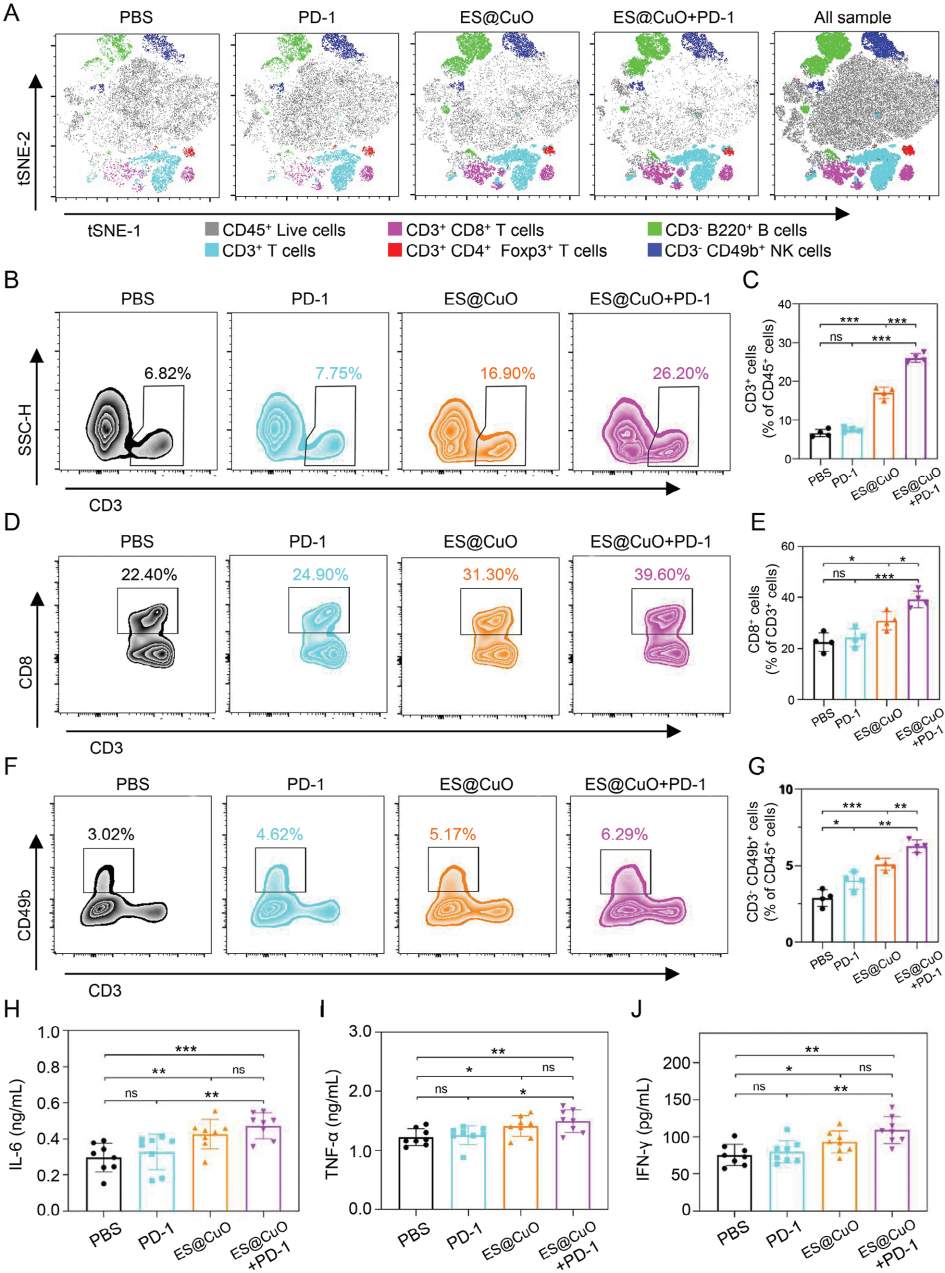
Assessment of antitumor immunity mediated by ES@CuO and PD‐1 combined therapy in vivo. A) T‐SNE analyses of the populations of tumor‐infiltrating lymphocytes, including CD45^+^ live lymphocytes, CD3^+^ T cells, CD3^+^CD8^+^ T cells, CD3^+^CD4^+^Foxp3^+^ Treg cells, CD3^−^B220^+^ B cells and CD3^−^CD49b^+^ NK cells, in the tumor tissues. B and C) Representative flow cytometry plots and the corresponding percentages of the CD3^+^ T‐cell population in tumor tissues from mice subjected to various treatments (*n* = 4). D and E) Representative flow cytometry plots and the corresponding percentages of the CD3^+^CD8^+^ T‐cell population in tumor tissues from mice subjected to various treatments (*n* = 4). F and G) Representative flow cytometry plots and the corresponding percentages of the CD3^−^CD49b^+^ NK cell population in tumor tissues from mice subjected to various treatments (*n* = 4). H–J) The serum levels of inflammatory factors (IL‐6, TNF‐α, and IFN‐γ) were measured via ELISAs (*n* = 8). Data are shown as the mean ± SD; p values were calculated using an unpaired, 2‐tailed Student's t test with Welch's correction; ns, not significant; *p < 0.05; **p < 0.01; ***p < 0.001.

To further explore the effect of ES@CuO+PD‐1 on the TME, we collected tumor tissues at the end of treatment to further investigate tumor‐infiltrating immune cells (Figure S18, Supporting Information). Subsequently, 2D t‐stochastic neighbor embedding (t‐SNE) projections of the immune landscape in the TME were constructed for visualization. The proportion of tumor‐infiltrating lymphocytes in the mice treated with ES@CuO+PD‐1 was greater than that in the other groups (**Figure** **6**A). Studies have reported that CD3^+^CD8^+^ cytotoxic T lymphocytes (CTLs) play a dominant role in eliminating tumor cells.^[^ ^35^, ^36^ ^]^ Flow cytometry analysis confirmed that the proportion of CTLs in the mice treated with ES@CuO+PD‐1 was greater than that in those treated with ES@CuO or PD‐1 alone (Figure 6B–E). Furthermore, an increase in the number of CTLs in the ES@CuO+PD‐1 group was detected by IHC staining for CD8^+^ cells in tumor tissues (Figure S19, Supporting Information). Regulatory T cells (Tregs), a class of immunosuppressive cells, are responsible for tumor cell immune escape.^[^ ^37^, ^38^ ^]^ Therefore, Treg infiltration in mouse tumors was further analyzed. The results showed that the proportion of Tregs in the mice treated with ES@CuO+PD‐1 was significantly lower than that in the control mice, while the proportion of CD3^+^CD4^+^ T cells did not change among the groups (Figure S20, Supporting Information). Natural killer (NK) cells are a subset of lymphocytes responsible for antitumor activity;^[^ ^39^, ^40^ ^]^ therefore, tumor‐infiltrating NK cells were also analyzed. The results demonstrated that more NK cells infiltrated the TME after treatment with ES@CuO+PD‐1 therapy (Figure 6F,G). The frequency of tumor‐infiltrating B lymphocytes, which can enhance antitumor immunity by presenting antigens and secreting inflammatory cytokines and antibodies,^[^ ^41^, ^42^ ^]^ was also markedly increased after ES@CuO+PD‐1 therapy (Figure S21, Supporting Information). Furthermore, the levels of interleukin 6 (IL‐6), tumor necrosis factor‐α (TNF‐α), and interferon‐γ (IFN‐γ), which further robustly activate immune responses,^[^ ^43^, ^44^ ^]^ substantially increased in the mice treated with ES@CuO+PD‐1 (Figure 6H–J). Together, these results confirmed that ES@CuO+PD‐1 therapy facilitated antitumor efficacy by effectively reprogramming the immunosuppressive TME.

### 2.11 Biosafety of Combined Therapy with ES@CuO and PD‐1 Therapy

To examine the biosafety of ES@CuO+PD‐1 in vivo, we collected blood and major organs from mice given the indicated treatments for further analysis. The major organs of the mice were assessed by H&E staining. Compared with PBS treatment, ES@CuO+PD‐1 combined therapy did not induce noticeable morphological changes or inflammation in major organs, including the heart, liver, spleen, lung, or kidney (Figure S22, Supporting Information). In addition, no significant hepatic or renal toxicity was induced by the combined therapy of ES@CuO+PD‐1, as indicated by the normal ranges of liver function markers, including alanine transaminase (ALT) and aspartate aminotransferase (AST), or kidney function markers, including cystatin C (Cys‐C) (Figure S23, Supporting Information). Overall, these data indicated the satisfactory biocompatibility of the combined therapy of ES@CuO+PD‐1 for antitumor treatment in vivo.

## 3 Conclusions

In summary, we constructed ES@CuO NPs as a pH‐responsive drug delivery nanoplatform for the codelivery of Cu^2+^ and ES. After cellular uptake, the ES@CuO microspheres resulted in the downregulation of FDX1 expression and the accumulation of aggregated DLAT, triggering tumor cell cuproptosis. Subsequently, ES@CuO, an ICD inducer, released many DAMPs, provoking cuproptosis‐mediated immune responses. In particular, the ES@CuO NPs displayed good tumor targeting and biosafety profiles. Given that melanoma is a malignancy, treatment with ES@CuO NPs alone slightly restricts melanoma progression. However, combined therapy with ES@CuO and PD‐1 synergistically remodeled the immunosuppressive TME by enhancing CTLs infiltration, significantly inhibiting the growth of B16 tumors in mice. Our findings demonstrate that these engineered nanoplatforms provide an effective paradigm for cuproptosis‐based antitumor therapy by increasing the efficacy of ICIs (Scheme 1). In addition, melanoma remains one of the most lethal malignancies because of its metastatic and invasive nature and unlimited replicative potential. Compared to conventional therapies for melanoma, immunotherapy has shown an improved prognosis. However, melanoma can gradually develop resistance to ICI‐based immunotherapy,^[^ ^7^, ^8^ ^]^ and detailed studies are needed to improve melanoma outcomes by effectively eliminating melanoma.

## 4 Experimental Section

### Materials

CuO, DSPE‐PEG_2000_‐NH2, fluorescein isothiocyanate (FITC), dimethyl sulfoxide (DMSO), and new indocyanine green (IR820) were obtained from Shanghai Aladdin Biochemical Technology Co., Ltd. Elesclomol (ES) was purchased from MedChemExpress. Phosphate‐buffered saline (PBS), fetal bovine serum (FBS), Dulbecco's modified Eagle's medium (DMEM), Roswell Park Memorial Institute (RPMI) 1640 medium, 0.25% trypsin‐EDTA and penicillin/streptomycin were obtained from Gibco. Annexin V‐FITC/PI Apoptosis Detection Kit (Cat: 556547) was purchased from BD Biosciences. FDX1 antibody (Cat: ab108257) and HMGB‐1 (Cat: ab79823) were purchased from Abcam. DLAT antibody (Cat: 9661), β‐Actin antibody (Cat: 3700), anti‐mouse IgG, HRP‐linked antibody (Cat: 7076), and anti‐rabbit IgG, HRP‐linked antibody (Cat: 7074) were purchased from Cell Signaling Technology, Inc. Cell Counting Kit‐8 (Cat: CK04), Cytotoxicity LDH Assay Kit (Cat: CK12), and ATP Assay Kit‐Luminescence (Cat: CK18) were obtained from Dojindo Laboratories. InVivoMAb anti‐mouse PD‐1 Antibody (Cat: BE0146) was obtained from Bio X Cell. TruStain FcX PLUS antibody (Cat: 156604), PE/Cyanine5 anti‐mouse CD45 antibody (Cat: 103110), PE anti‐mouse CD3 antibody (Cat: 100206), FITC anti‐mouse CD4 antibody (Cat: 100406), APC/Cyanine7 anti‐mouse CD8a antibody (Cat: 100714), B220‐Brilliant Violet 785 (Cat: 103246), and APC anti‐mouse CD49b antibody (Cat: 108910) were obtained from BioLegend, Inc. LIVE/DEAD Fixable Dead Cell Stain Kit (Cat: L34955), PE‐Cyanine7 FOXP3 Monoclonal Antibody (Cat: 25‐5773‐82), Ki‐67 Monoclonal Antibody (Cat: 14‐5698‐82), DAPI (Cat: D3571), Hoechst 33 342 (Cat: H1399), Goat anti‐Mouse IgG (H+L) Cross‐Adsorbed Secondary Antibody, Alexa Fluor 488 (Cat: A‐11001) were obtained from Invitrogen. BCA Protein Quantification Kit (Cat: 20201) and Precast Protein Plus Gel (4%–12%, 15 wells) (Cat: 36255) were obtained from Yeasen Biotechnology (Shanghai) Co., Ltd. Calcein‐AM/PI Live/Dead Viability Assay Kit, RIPA, Protease Inhibitor Cocktail, LysoTracker Red, Crystal violet, DCFH‐DA, Dil, and TMB were purchased from Shanghai Beyotime Biotechnology Co., Ltd. Enzyme‐linked immunosorbent assay (ELISA) kits for HMGB‐1, IL‐6, TNF‐α, IFN‐γ, ALT, AST and Cys‐C were obtained from Jiangsu Meimian Industrial Co., Ltd.

### Synthesis of ES@CuO NPs

ES (10 mg in 1 mL DMSO) and CuO NPs (10 mg in 9 mL ultrapure water) were stirred for 12 h at room temperature. Then, 10 mg DSPE‐PEG_2000_‐NH2 was added to the mixture, and the mixture was stirred for 12 h at room temperature. After stirring, the precipitate was recovered by centrifugation (12 000 g, 15 min), and the collected residue was washed with ultrapure water three times.

### Synthesis of FTIC@CuO NPs

CuO NPs (5 mg in 4.5 mL ultrapure water) and FITC (5 mg in 0.5 mL DMSO) were stirred for 12 h in a dark environment, 5 mg DSPE‐PEG_2000_‐NH2 was added to the mixed solution, and the mixture was stirred for 12 h at room temperature. The suspension was centrifuged (12 000 g, 15 min) and washed with ultrapure water five times until the supernatant was free of fluorescent dye.

### Synthesis of IR820@CuO NPs

5 mg CuO NPs and 5 mg IR820 were dispersed in 5 mL ultrapure water. The mixture was stirred for 12 h in a dark environment. Then, 5 mg DSPE‐PEG_2000_‐NH2 was added to the suspension, and the mixture was stirred for 12 h at room temperature. The mixed solution was centrifuged (12 000 g, 15 min) and washed with ultrapure water five times until the supernatant was free of fluorescent dye.

### Material Characterization

The morphology and elemental composition of the nanoparticles were evaluated via transmission electron microscopy (TEM) (JEOL, JEM‐F200, Japan). The UV‒vis absorption spectrum was measured using a UV‒vis spectrometer (MAPADA, Shanghai). Dynamic light scattering (DLS) and surface zeta potential were characterized by a Zetasizer Nano Analyzer (Malvern, U.K.). Fourier transform infrared (FTIR) spectra were recorded on an FTIR spectrometer (Thermo Scientific Nicolet iS20, USA) with wavenumbers ranging from 400 to 4000 cm^−1^. X‐ray photoelectron spectroscopy (XPS) was performed using a Thermo Scientific K‐Alpha. X‐ray diffraction (XRD) patterns were obtained using a Rigaku Ultima IV instrument.

### Drug Loading Efficiency

The absorbance of ES standard concentration and free ES remaining in the supernatant was measured by a UV‒Vis spectrophotometer at 390 nm. The drug loading content (LC%) of ES was calculated using the following formula: LC% = M_Encapsulated ES_/M_Initial ES_ ×100%.

### pH‐Responsive Ability

In brief, ES@CuO (100 µg mL^−1^) NPs were added to different PBS buffers (pH = 4.5, 5.5, and 7.5) and gently shaken at 100 rpm at room temperature. The solution was centrifuged (12 000 g for 20 min) at the indicated timepoints, and the released free Cu^2+^ and ES in the supernatants were quantified by ICP‒MS (Agilent 7700) and HPLC (Agilent HPLC 1260), respectively.

### Cell Culture

The B16 mouse melanoma cell line was obtained from the Cell Bank of the Chinese Academy of Sciences. B16 cells were cultured in RPMI‐1640 medium supplemented with 10% FBS and 1% penicillin/streptomycin in a Thermo Scientific cell incubator at 37 °C under 5% CO_2_.

### Cellular Uptake

A total of 1 × 10^6^ B16 cells were seeded in a 6‐well plate and incubated overnight, and then the cells were treated with FITC‐labeled CuO (FITC@CuO) NPs (1 µg mL^−1^) for 6 h. After the incubation, the cells were washed with PBS three times, and the intracellular intensity of FITC was analyzed via a flow cytometer (Beckman Coulter CytoFLEX). For subcellular localization, 1 × 10^5^ B16 cells were seeded in a confocal glass bottom dish and incubated with FITC@CuO (1 µg mL^−1^) for 6 h. Then, the cells were stained with Hoechst 33342 and LysoTracker Red for 15 min and observed with a confocal laser scanning microscope (Leica, Germany).

### Intracellular Cu Content Assay

A total of 1 × 10^6^ B16 cells were seeded in 6‐well plates and incubated overnight. FITC@CuO (1 µg mL^−1^) was incubated with B16 cells for 6 h, after which the cells were washed with PBS three times. Then, the cell sample was digested with 2% nitric acid (0.2 mL) at 65 °C for 1 h.^[^ ^18^ ^]^ The intracellular Cu concentration in the cell lysis solution was determined via ICP‒MS analysis.

### TMB Experiment

⋅OH, generation was investigated by using TMB as a probe. Cu^2+^ (at a final concentration of 10 µg mL^−1^) was added to the TMB solution (at a final concentration of 0.5 mM) in PBS buffer at pH 5.0, after which the H_2_O_2_ solution (at a final concentration of 8 mM) was added. After 5 min of incubation, the absorption spectrum of the solution was recorded by a UV−vis spectrophotometer.

### Cytotoxicity Assay

B16 cells were seeded in a 96‐well plate (1 × 10^4^ cells well^−1^) and incubated overnight. Then, the cells were treated with the indicated NPs at different concentrations for 24 h. Afterward, the cells were incubated with Cell Counting Kit‐8 (CCK‐8) solution for another 3 h and analyzed using a microplate reader (Tecan, Switzerland) at a wavelength of 450 nm.

### Cell Morphology

B16 cells were seeded into glass‐bottom cell culture dishes (1 × 10^5^ cells dish^−1^) and incubated overnight. Then, the cells were incubated with PBS, CuCl_2_ (5 µM), ES (50 nM), CuCl_2_+ES (5 µM+ 50 nM), CuO (0.4 µg mL^−1^) or ES@CuO (0.4 µg mL^−1^) for 4 h. The morphology of the cells was further observed by an inverted fluorescence microscope (Leica, Germany).

### Colony Formation

B16 cells (800 cells well^−1^) were seeded in 12‐well plates for 72 h in an incubator (37 °C, 5% CO_2_). B16 cells were subsequently treated with PBS, CuCl_2_ (5 µM), ES (50 nM), CuCl_2_+ES (5 µM+ 50 nM), CuO (0.4 µg mL^−1^) or ES@CuO (0.4 µg mL^−1^) and then incubated for another 48 h. After treatment, the cells were fixed with 4% paraformaldehyde for 10 min and stained with 0.2% crystal violet for 10 min. Images were captured by camera after washing with ultrapure water several times.

### Apoptosis Assay

B16 cells were seeded into 6‐well plates (5 × 10^5^ cells well^−1^) and incubated overnight. Then, the cells were incubated with PBS, CuCl_2_ (5 µM), ES (50 nM), CuCl_2_+ES (5 µM+ 50 nM), CuO (0.4 µg mL^−1^) or ES@CuO (0.4 µg mL^−1^) for 24 h. The collected cells were stained with an Annexin‐FITC and PI Apoptosis Detection Kit (BD Biosciences) and analyzed by flow cytometry (Beckman Coulter, USA).

### LDH and ATP Assays

B16 cells were seeded into 96‐well plates (1 × 10^4^ cells well^−1^) and incubated overnight. Then, the cells were treated with PBS, CuCl_2_ (5 µM), ES (50 nM), CuCl_2_+ES (5 µM+ 50 nM), CuO (0.4 µg mL^−1^) or ES@CuO (0.4 µg mL^−1^) for 24 h. After that, the extracellular LDH and intracellular ATP concentrations were determined by LDH and ATP Assay Kit‐Luminescence (Dojindo, Japan), respectively, according to the manufacturer's protocol.

### Immunofluorescence Analysis of HMGB‐1 Expression

B16 cells (1 × 10^5^) were seeded in poly‐L‐lysine‐treated confocal dishes and incubated overnight. Then, the cells were treated with PBS, CuCl_2_ (5 µM), ES (50 nM), CuCl_2_+ES (5 µM+ 50 nM), CuO (0.4 µg mL^−1^) or ES@CuO (0.4 µg mL^−1^) for 24 h. After incubation, the cells were fixed with 4% PFA and stained with an anti‐HMGB‐1 antibody. DAPI was used to stain nuclei, and the expression of HMGB‐1 was observed via CLSM.

### Immunofluorescence of DLAT Oligomerization

B16 cells were seeded in glass‐bottom cell culture dishes (1 × 10^5^ cells dish^−1^) and incubated overnight. The cells were then treated with PBS, CuCl_2_ (5 µM), ES (50 nM), CuCl_2_+ES (5 µM+ 50 nM), CuO (0.4 µg mL^−1^) or ES@CuO (0.4 µg mL^−1^) for 2 h. Before 4% paraformaldehyde fixation, the cells were incubated with MitoTracker Red CMXRos for 30 min. Fixed cells were incubated with a DLAT antibody (dilution 1:200) at 4 °C overnight and subsequently incubated with an Alexa Fluor 488‐conjugated anti‐mouse secondary antibody at room temperature for 1 h.^[^ ^18^ ^]^ Then, the cells were stained with DAPI at room temperature for 15 min and detected by a confocal laser scanning microscope (Leica, Germany).

### Knockdown of FDX1 Expression

Lentiviral particles were produced in HEK293T cells for lentiviral infection to inhibit FDX1 gene expression (shFDX1 target sequence: GCTCTACTTGTCATCTTATCT) in B16 cells according to the Addgene protocols (https://www.addgene.org/protocols).

### Western Blot

B16 cells were seeded into 6‐well plates at a density of 1 × 10^6^ cells well^−1^ for 12 h. Subsequently, the cells were treated with PBS, CuCl_2_ (5 µM), ES (50 nM), CuCl_2_+ES (5 µM+ 50 nM), CuO (0.4 µg mL^−1^) or ES@CuO (0.4 µg mL) for 2 h. Then, the cells were collected and lysed with radioimmunoprecipitation assay (RIPA) lysis buffer, followed by sonication. The protein samples were extracted by centrifugation (12 000 rpm, 15 min), and a BCA protein assay kit was used to determine the concentration of the proteins. The protein samples were separated via 12% sodium dodecyl sulfate‒polyacrylamide gel electrophoresis (SDS‒PAGE) and transferred to polyvinylidene fluoride (PVDF) membranes. After blocking with 5% nonfat milk for 1 h at room temperature, the PVDF membranes were incubated with an FDX1 antibody (1:1000 dilution) overnight at 4 °C. Subsequently, the PVDF membranes were incubated with the corresponding horseradish peroxidase (HRP)‐conjugated antibody for 2 h at room temperature. The PVDF membranes were incubated with an enhanced chemiluminescence (ECL) reagent (Millipore), and images were acquired with a Bio‐Rad Gel Doc XR^+^ Gel Documentation System. Western blot data were quantified with ImageJ software (NIH, Bethesda, MD).

### Mouse Tumor Models

Male C57BL/6 N mice aged 4 weeks were purchased from Vital River Laboratory Animal Technology Co., Ltd. (Shanghai) and raised in specific‐pathogen‐free (SPF) animal rooms. All animal experiments were performed with the permission of the Institutional Animal Care and Use Committee of Wenzhou Institute, University of Chinese Academy of Sciences (Approval number: WIUCAS22110103). To establish the B16 tumor‐bearing model, 50 µL of B16 cell suspension (1 × 10^6^) was subcutaneously injected into the right flanks of the mice. Five days later, when the tumor volume reached ≈80 mm^3^, the mice were randomly divided into four groups (*n* = 5) and intravenously administered PBS, CuO (100 µg mouse^−1^), ES (95 µg/mouse), or ES@CuO (100 µg mouse^−1^) every 2 days for a total of 3 times. Tumor size and body weight were measured every other day. The tumor volume was calculated using the formula: tumor volume (mm^3^) = (width^2^ × length)/2. At the end of the observation period (days 12), the mice were sacrificed, and the tumors were harvested, weighed, and analyzed by hematoxylin and eosin (H&E) and immunohistochemistry (IHC) staining. For survival studies, mice were euthanized once the tumor volume exceeded 1500 mm^3^. Survival data from each group were analyzed using the Kaplan‒Meier method, and the significance of the differences was tested via the log‐rank test (Mantel–Cox).

PD‐1+ES@CuO combined immunotherapy. A B16 tumor model was established by subcutaneously injecting B16 cells (1 × 10^6^) into the right flank of mice on day 0. On days 5, when the tumor volume reached ≈80 mm^3^, the mice were randomly divided into four groups: PBS, PD‐1, ES@CuO, and ES@CuO+PD‐1 (*n* = 5). The mice were intraperitoneally administered an anti‐PD‐1 antibody (100 µg mouse^−1^) and intravenously injected with ES@CuO (100 µg mouse^−1^) on days 5, 7, and 9. After the treatments, mouse blood, major organs (heart, liver, spleen, lung, and kidney), and tumor tissues were collected for subsequent experiments.

In vivo fluorescence imaging. B16 tumor cells (1 × 10^6^) were subcutaneously injected into the right flank of male C57BL/6 N mice. When the tumor volume was ≈200 mm^3^, the mice were randomly divided into two groups (*n* = 3). Then, IR820 (2 µg mouse^−1^) and IR820@CuO (10 µg mouse^−1^) were injected into the mice via the tail vein. At different time points (0, 1, 6, 12, and 24 h), the mice were anesthetized and imaged by an IVIS Lumina III imaging system (Perkin Elmer, USA; excitation filter: 780 nm; emission filter: 820 nm).

### Tumor‐Infiltrating Lymphocyte Analysis

To analyze the infiltrating lymphocytes, primary tumors were collected to obtain lymphocytes. Tumor tissues were cut into small pieces and digested with RPMI 1640 medium containing 1 mg mL^−1^ collagenase IV, 0.1 mg mL^−1^ hyaluronidase, 0.1 mg mL^−1^ DNase I, and 2% FBS at 37 °C for 30 min. Digested tumors were dissociated with gentleMACS Dissociator (Miltenyi Biotech) and filtered through a 40‐µm strainer to obtain a single‐cell suspension. The cells were purified by Percoll gradient centrifugation and blocked with Mice TruStain FcX (Fc Receptor Blocking Solution, BioLegend) on ice for 10 min. The cells were stained with a LIVE/DEAD Fixable Violet Kit (Invitrogen) and then stained with the following anti‐mouse antibodies: CD45‐PE/Cyanine5, CD3‐PE, CD4‐FITC, CD8‐APC/Cyanine7, CD49b‐APC, B220‐Brilliant Violet 785, and FOXP3‐PE‐Cyanine7 according to the manufacturer's methods. Finally, the stained cells were analyzed via a flow cytometer (Beckman Coulter).^[^ ^45^ ^]^ Data analysis was conducted using FlowJo software (BD, USA). The types of immune cells were subjected to density clustering after they were identified using nonlinear dimensionality reduction [t‐distributed stochastic neighbor embedding (t‐SNE)].

### Histopathological Analysis

The collected tumors and organs were fixed in 4% paraformaldehyde solution for 24 h and subsequently dehydrated, paraffin‐embedded, sectioned, and stained with H&E. For IHC staining, the sections were repaired with sodium citrate buffer (pH = 6.0) and then blocked with 3% H_2_O_2_ for 15 min at room temperature. After permeabilization with 0.5% Triton X‐100, the sections were blocked with goat serum at room temperature for 1 h and incubated with the corresponding primary antibody at 4 °C overnight. After that, the sections were incubated with an anti‐mouse/rabbit secondary antibody (Dako REAL EnVision Detection System) for 1 h at room temperature. Finally, the sections were stained with 3,3′‐diaminobenzidine (DAB) for color development.^[^ ^45^ ^]^ The slides were then counterstained with hematoxylin, dehydrated, and imaged under an optical microscope (Nikon, Japan).

### Enzyme‐Linked Immunosorbent Assay (ELISA)

After various treatments, the collected blood samples were naturally solidified at 4 °C for 2 h and centrifuged at 2000 g for 20 min at 4 °C to obtain the blood serum. Blood biochemistry and cytokine levels were determined using ELISA kits according to the manufacturer's protocol.

### Statistical Analysis

Data are presented as the mean ± standard deviation (SD). The specific statistical sample size for each experiment is shown in the figure legend. Statistical analyses were conducted using GraphPad Prism 8 (GraphPad Software, San Diego, CA). Student's t test was used to determine the significance of differences between two groups. Survival analysis was determined by Kaplan‒Meier analysis followed by the log‐rank test. A difference of *p < 0.05 was considered to indicate statistical significance, and **p < 0.01 and ***p < 0.001 were considered to indicate high significance.

## Conflict of Interest

The authors declare no conflict of interest.

## Supporting information

Supporting Information

## Data Availability

The data that support the findings of this study are available in the supplementary material of this article.
