# Supplementary material for: Elesclomol Loaded Copper Oxide Nanoplatform Triggers Cuproptosis to Enhance Antitumor Immunotherapy
Source: Adv Sci (Weinh). 2024 Mar 2;11(18):2309984. doi: 10.1002/advs.202309984 (PMC11095170; doi:10.1002/advs.202309984)
Supplement: Supplementary file 1 — Supporting Information [file ADVS-11-2309984-s001.pdf]

ADVANCED SCIENCE
Open Access

Supporting Information

for *Adv. Sci.*, DOI 10.1002/advs.202309984

Elesclomol Loaded Copper Oxide Nanoplatform Triggers Cuproptosis to Enhance Antitumor Immunotherapy

Xufeng Lu, Xiaodong Chen, Chengyin Lin, Yongdong Yi, Shengsheng Zhao, Bingzi Zhu, Wenhai Deng, Xiang Wang, Zuoliang Xie, Shangrui Rao, Zhonglin Ni, Tao You, Liyi Li, Yingpeng Huang, Xiangyang Xue, Yaojun Yu, Weijian Sun* and Xian Shen**

Supporting information

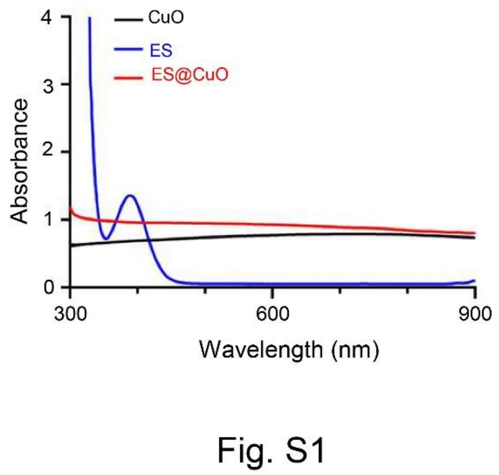

Fig. S1. UV–vis absorption spectra of CuO (in ultrapure water), ES (in DMSO), and ES@CuO (in ultrapure water).

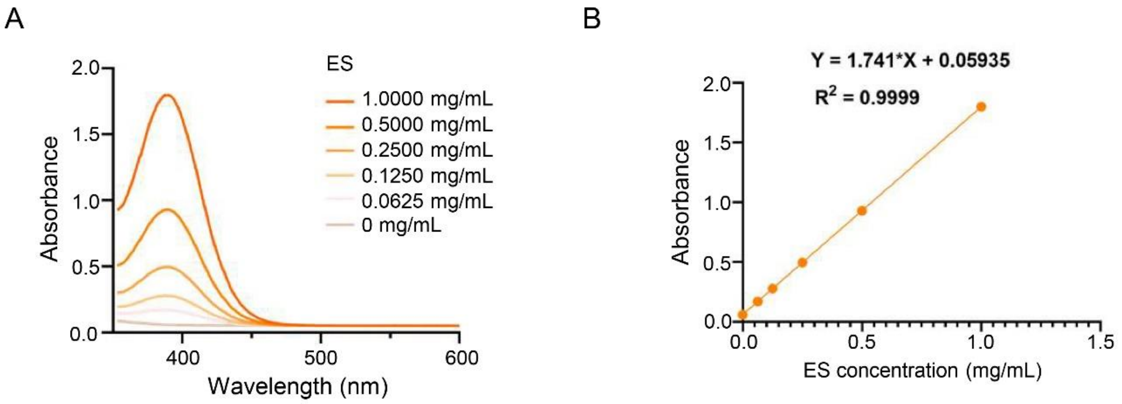

Fig. S2

Fig. S2. UV–vis absorption spectra of ES.

(A) UV–vis absorption spectra of ES at different concentrations (in DMSO). (B) The standard curve of the absorption intensity (390 nm) of ES as a function of concentration.

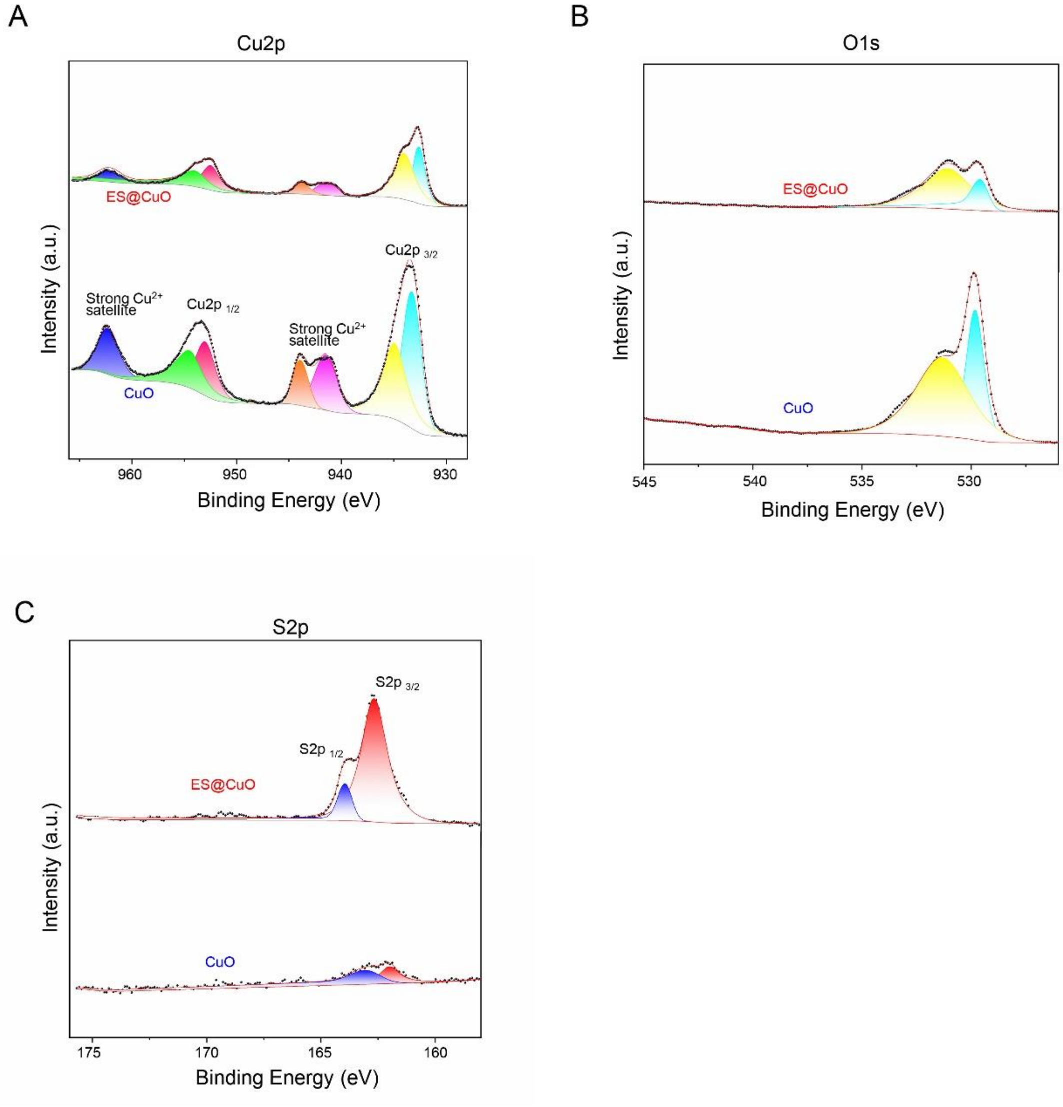

Fig. S3

Fig. S3. High-resolution Cu2p (A), O1s (B), and S2p (C) XPS spectra of CuO and ES@CuO.

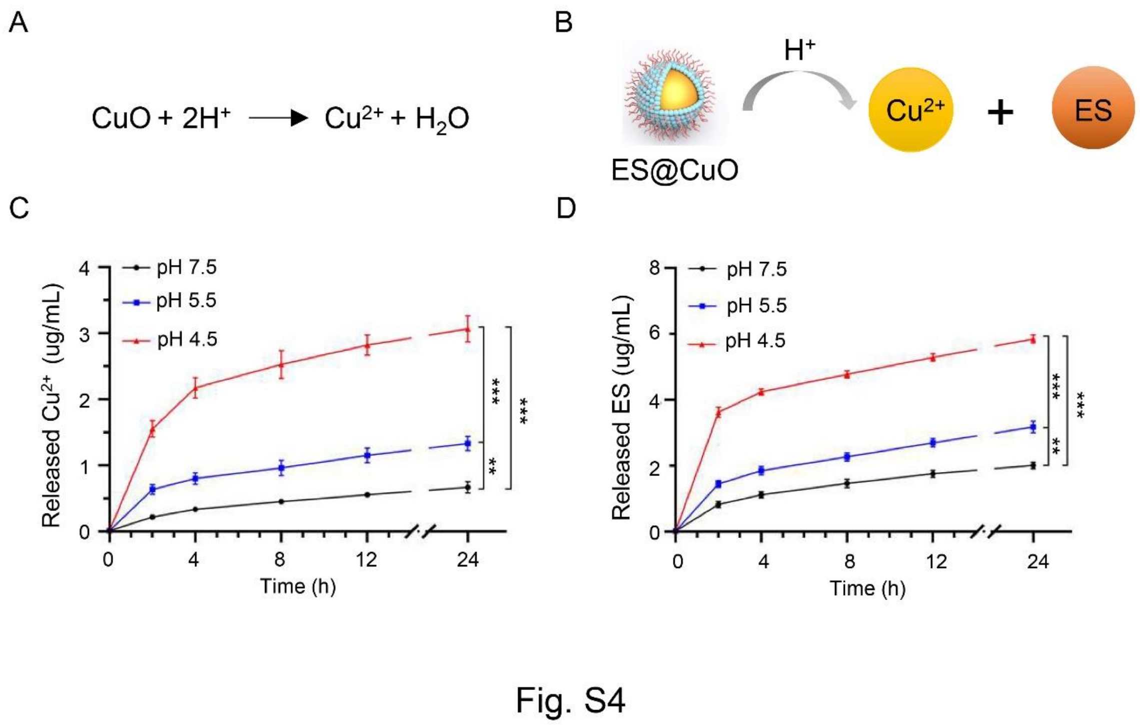

Fig. S4. pH response of ES@CuO.

(A) and (B) Schematic illustration of the pH-responsive degradation of ES@CuO and the corresponding release of Cu^{2+} and ES. (C) Cumulative Cu^{2+} release from ES@CuO (100 µg/mL) in PBS solution at pH 4.5, 5.5, and pH 7.5 ($n = 3$). (D) Cumulative release of ES from ES@CuO (100 µg/mL) in PBS solution at pH 4.5, 5.5 and 7.5 ($n = 3$). Data are shown as the mean ± SD; p values were calculated using an unpaired, 2-tailed Student's t test with Welch's correction; $^{**}p < 0.01$; $^{***}p < 0.001$.

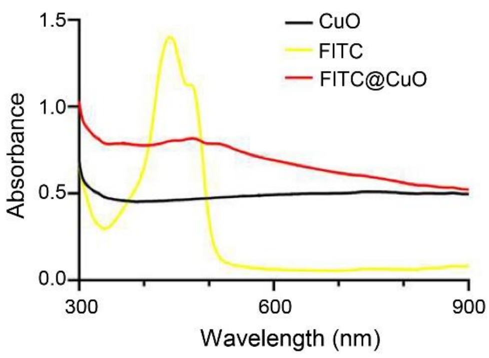

Fig. S5

Fig. S5. UV–vis absorption spectra of CuO (in ultrapure water), FITC (in ultrapure water), and FITC@CuO (in ultrapure water).

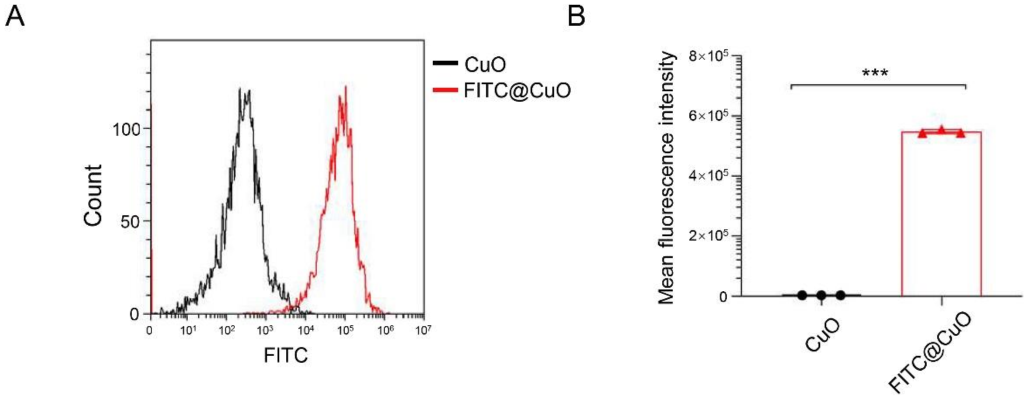

Fig. S6

Fig. S6. The intracellular uptake of FITC@CuO NPs.

(A) Flow cytometry analysis of B16 tumor cells treated with the indicated NPs for 6 h and (B) the corresponding mean intracellular fluorescence intensity ($n = 3$). Data are shown as the mean ± SD; p values were calculated using an unpaired, 2-tailed Student's t test with Welch's correction; $^{***}p < 0.001$.

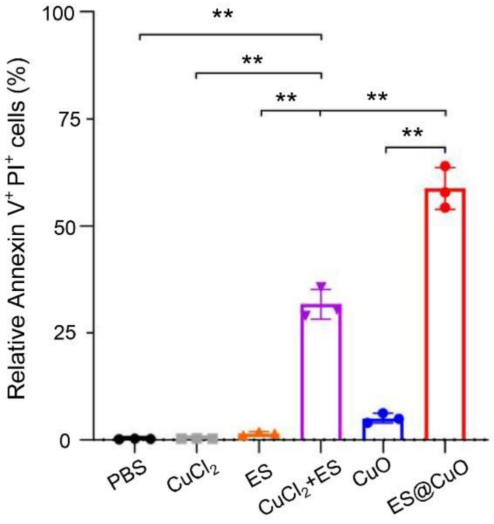

Fig. S7

Fig. S7. Quantification of PI^+/Annexin V-$FITC^+$ B16 cells after the indicated treatment (n = 3). Data are shown as the mean ± SD; p values were calculated using an unpaired, 2-tailed Student's t test with Welch's correction; $^{**}p < 0.01$.

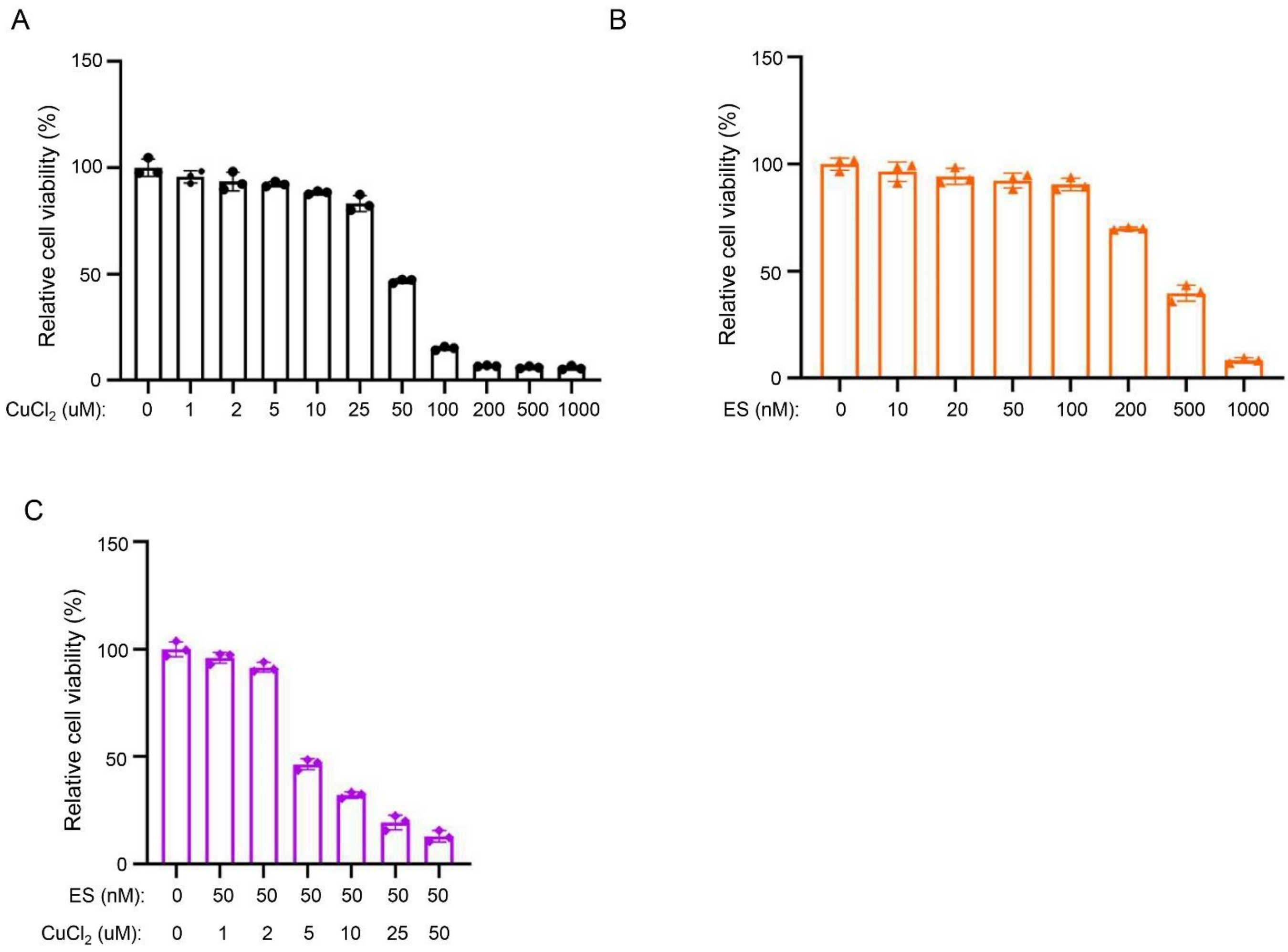

Fig. S8. Cell viability of B16 cells treated with various concentrations of $CuCl_2$ (A), ES (B), and $CuCl_2$+ES (C) for 24 h (n = 3). Data are shown as the mean ± SD.

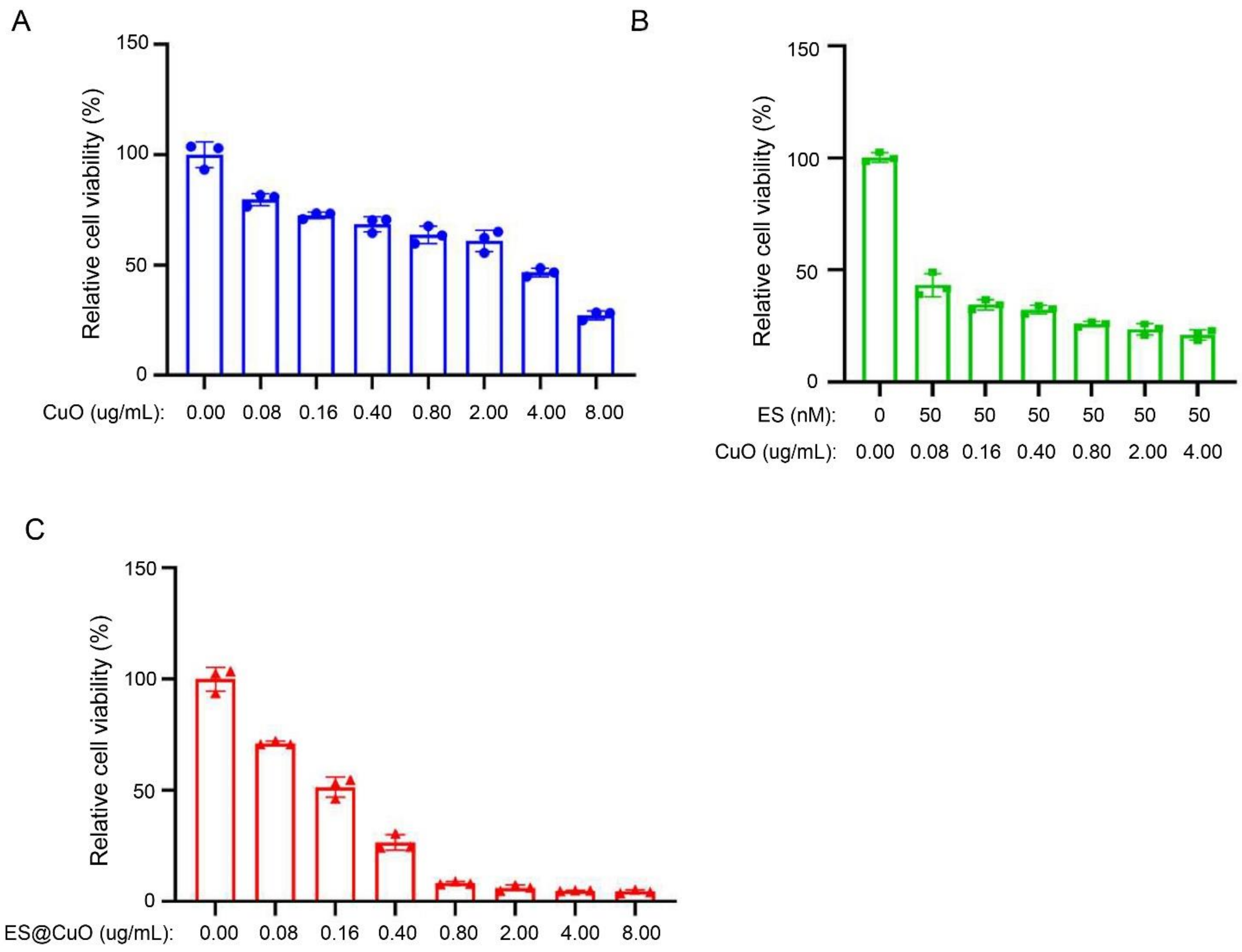

Fig. S9

Fig. S9. Cell viability of B16 cells treated with various concentrations of CuO (A), CuO+ES (B), or ES@CuO (C) for 24 h (n = 3). 0.40 μg/mL CuO (79.55 g/mol) ≈ 5 μM CuO. Data are shown as the mean ± SD.

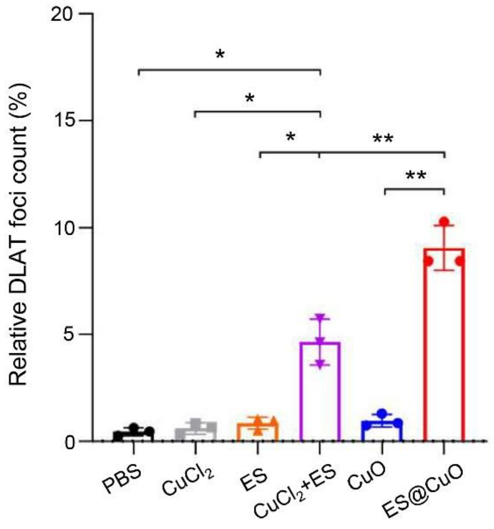

Fig. S10

Fig. S10. Quantification of DLAT foci in B16 cells after the indicated treatment (n = 3). Data are shown as the mean ± SD; p values were calculated using an unpaired, 2-tailed Student's t test with Welch's correction; *$p < 0.05$; **$p < 0.01$.

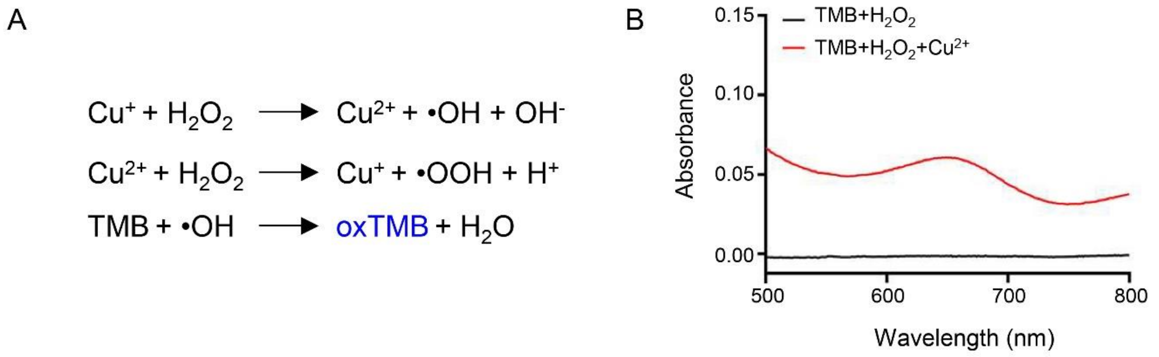

Fig. S11

Fig. S11. Copper iron-induced ·OH generation via a Fenton-like reaction. (A) Schematic illustration of the detection of ·OH generation from Cu^{2+} by TMB assays. (B) UV–vis spectra of TMB solutions (0.5 mM) incubated with H_2O_2 (8 mM) and Cu^{2+} (10 µg/mL) for 5 min.

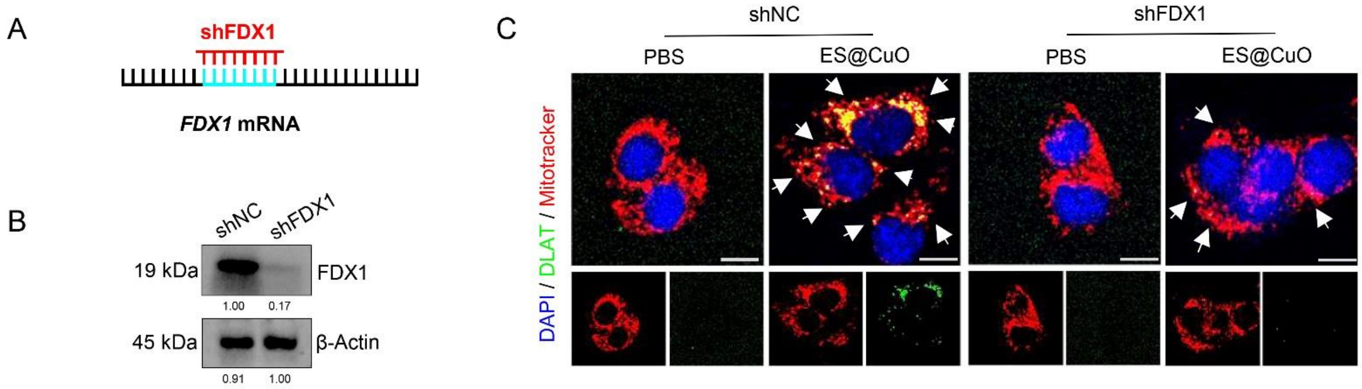

Fig. S12

Fig. S12. FDX1 knockdown efficiently inhibited the ES@CuO-induced oligomerization of DLAT in B16 cells.

(A) Illustration of the shRNA used for the FDX1 knockdown assay. (B) shFDX1 efficiently inhibited FDX1 expression in B16 cells. (C) Representative confocal microscopy images of DLAT aggregation in B16 cells after the indicated treatment. The white arrows indicate DLAT aggregation. Scale bars: 10 μm.

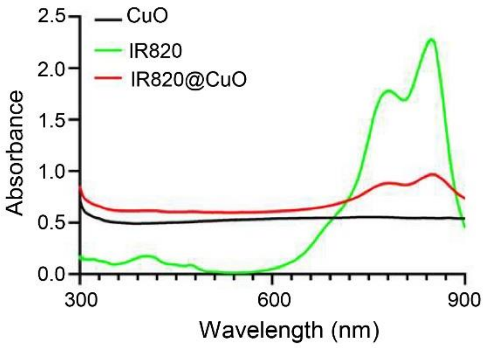

Fig. S13

Fig. S13. UV–vis absorption spectra of CuO (in ultrapure water), IR820 (in ultrapure water), and IR820@CuO (in ultrapure water).

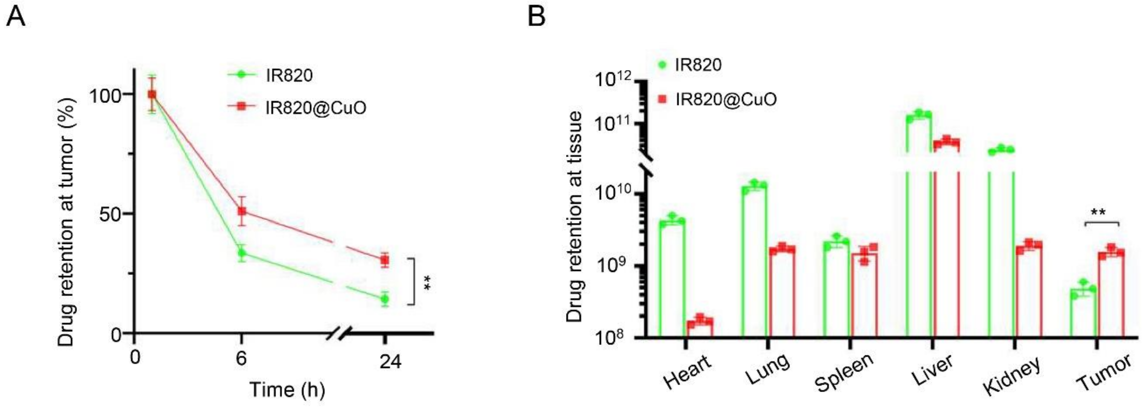

Fig. S14

Fig. S14. *Ex vivo* fluorescence intensity in tumors and major organs.

(A) and (B) Quantitative analysis of *ex vivo* IR820 fluorescence intensity in tumor sites and major organs (heart, lung, liver, spleen, and kidney) collected at the indicated time points after intravenous injection (n = 3). Data are shown as the mean ± SD; p values were calculated using an unpaired, 2-tailed Student's t test with Welch's correction; $^{**}p < 0.01$.

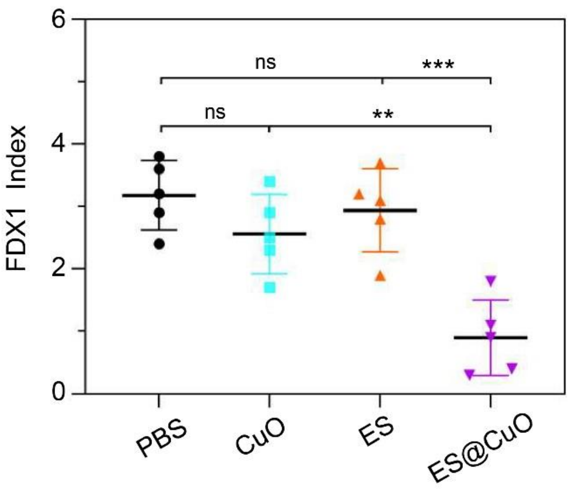

Fig. S15

Fig. S15. The corresponding quantification of FDX1 expression in tumor tissues after various treatments (n = 5). Data are shown as the mean ± SD; p values were calculated using an unpaired, 2-tailed Student's t test with Welch's correction; ns, not significant; $^{**}p < 0.01$; $^{***}p < 0.001$.

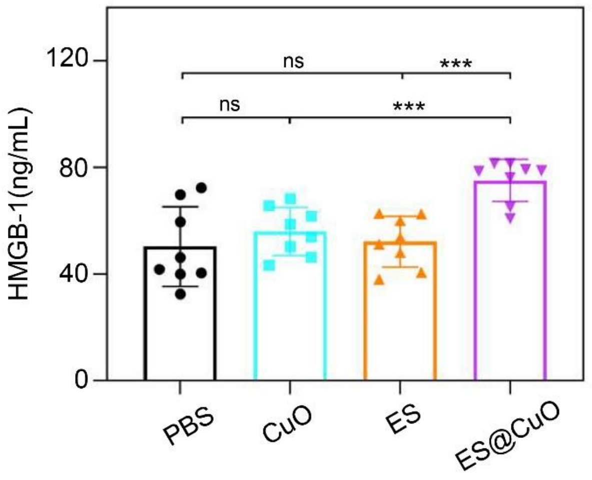

Fig. S16

Fig. S16. The serum level of HMGB-1 after different treatments was measured via ELISA (n = 8). Data are shown as the mean ± SD; p values were calculated using an unpaired, 2-tailed Student's t test with Welch's correction; ns, nonsignificant; ***$p < 0.001$.

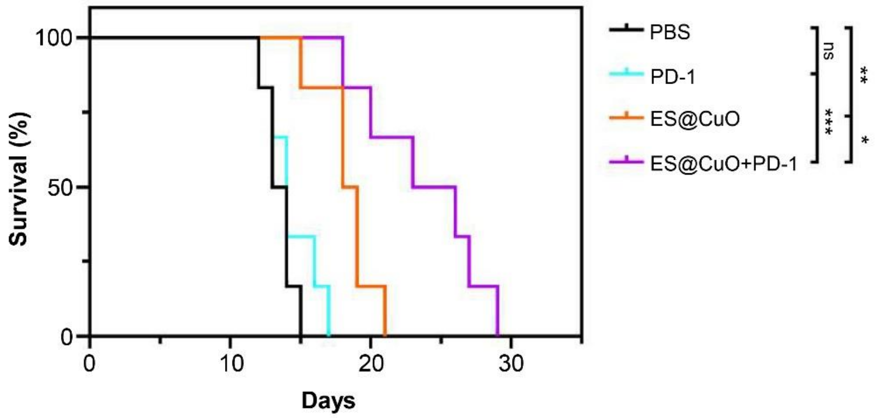

Fig. S17

Fig. S17. Survival curve of B16 tumor-bearing mice after different treatments (n = 6). Survival analysis was determined by Kaplan–Meier analysis followed by the log-rank test; ns, not significant; *$p < 0.05$; **$p < 0.01$; ***$p < 0.001$.

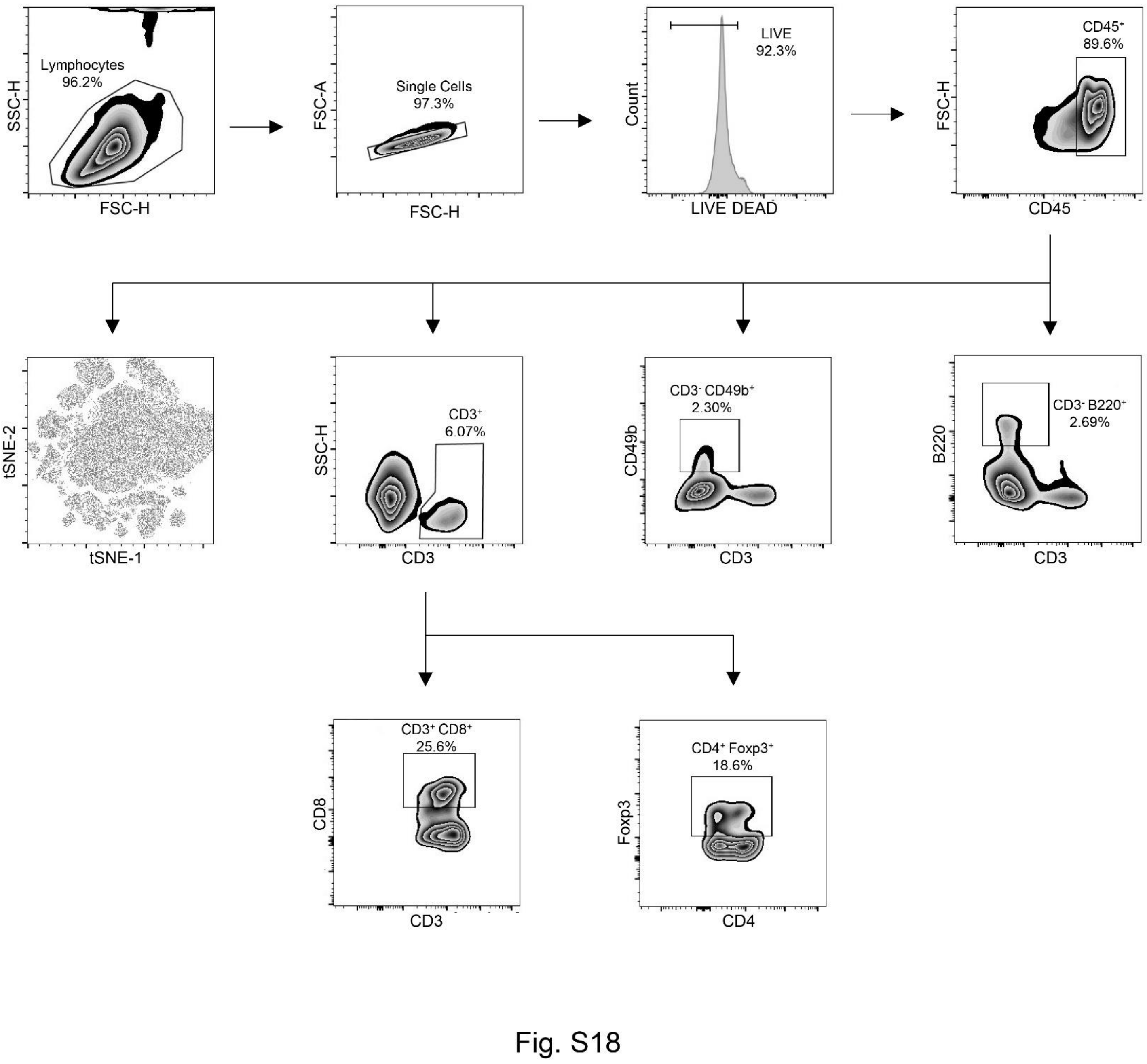

Fig. S18. The gating strategy for tumor-infiltrating lymphocytes in tumor tissues after various treatments.

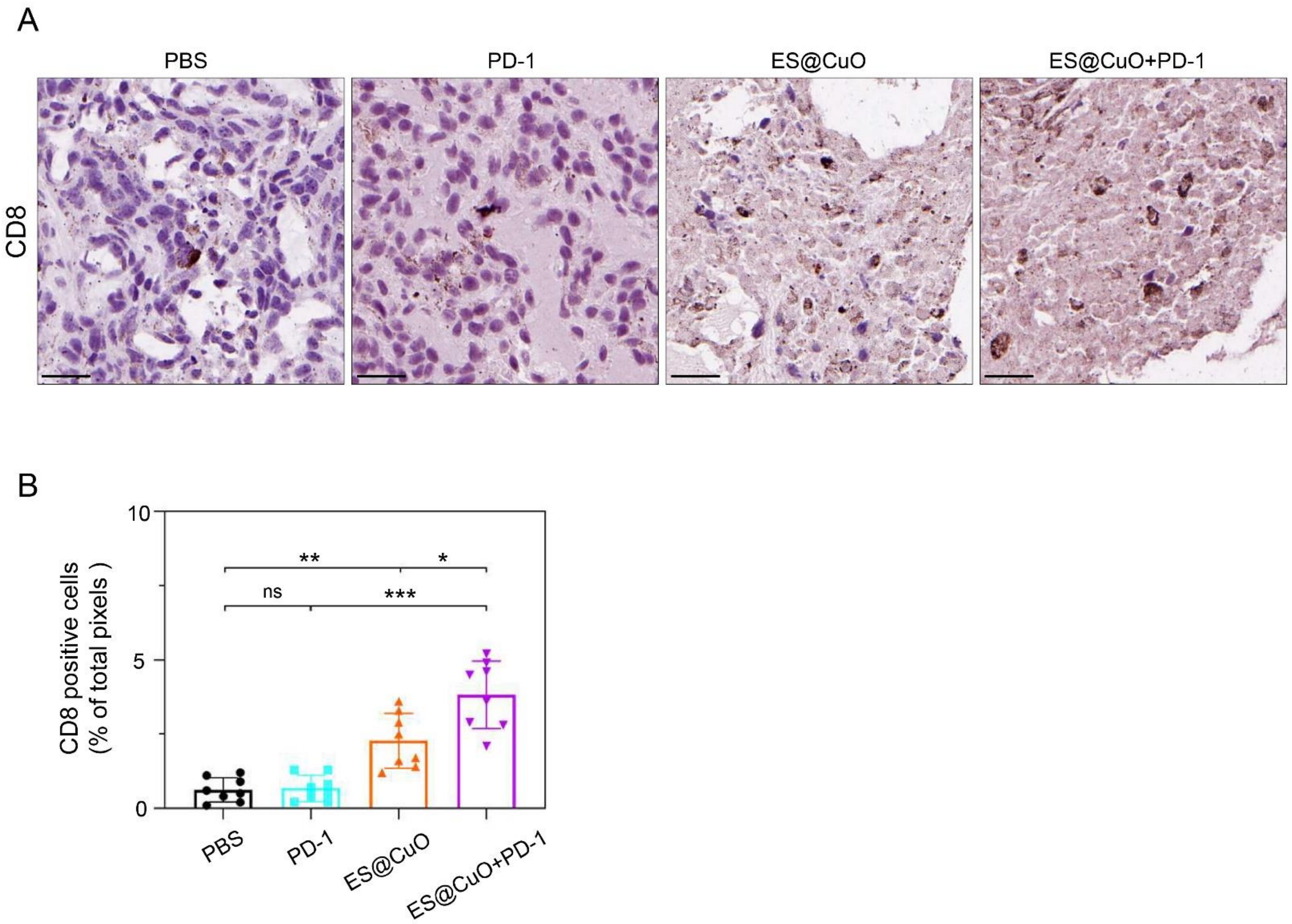

Fig. S19

Fig. S19. Representative IHC staining images and the corresponding quantification of CD8 expression in tumor tissues after various treatments (n = 8). Scale bar: 25 μm. Data are shown as the mean ± SD; p values were calculated using an unpaired, 2-tailed Student's t test with Welch's correction; ns, not significant; *$p < 0.05$; **$p < 0.01$; ***$p < 0.001$.

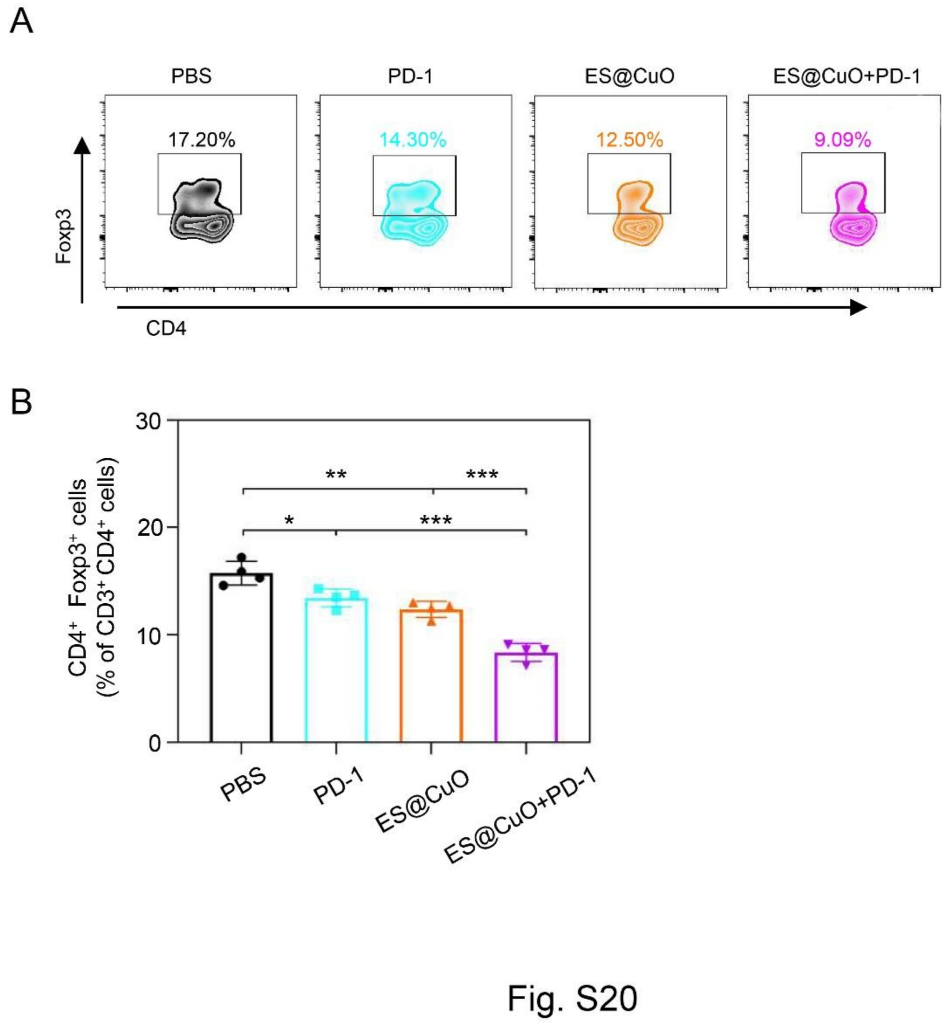

Fig. S20. Representative flow cytometry plots and the corresponding percentages of the $CD3^{+}CD4^{+}Foxp3^{+}$ Treg cell population in tumor tissues from mice subjected to various treatments (n = 4). Data are shown as the mean ± SD; p values were calculated using an unpaired, 2-tailed Student's t test with Welch's correction; *p < 0.05; **p < 0.01; ***p < 0.001.

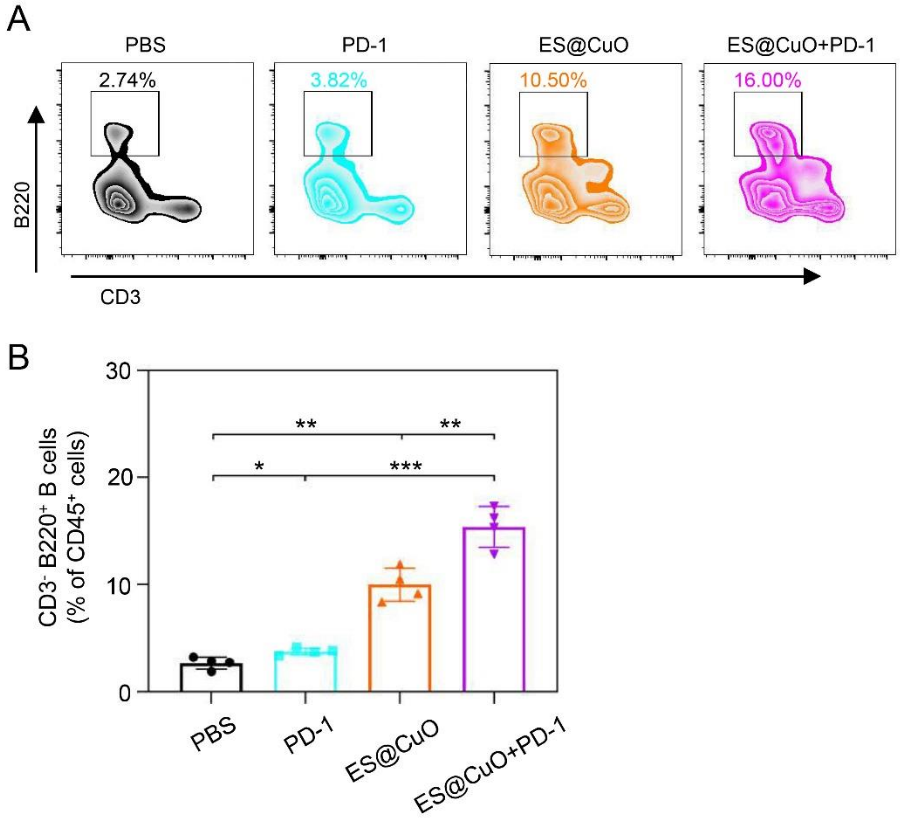

Fig. S21

Fig. S21. Representative flow cytometry plots and the corresponding percentages of the $CD3^{-}B220^{+}$ B-cell population in tumor tissues from mice given various treatments ($n = 4$). Data are shown as the mean ± SD; p values were calculated using an unpaired, 2-tailed Student's t test with Welch's correction; *$p < 0.05$; **$p < 0.01$; ***$p < 0.001$.

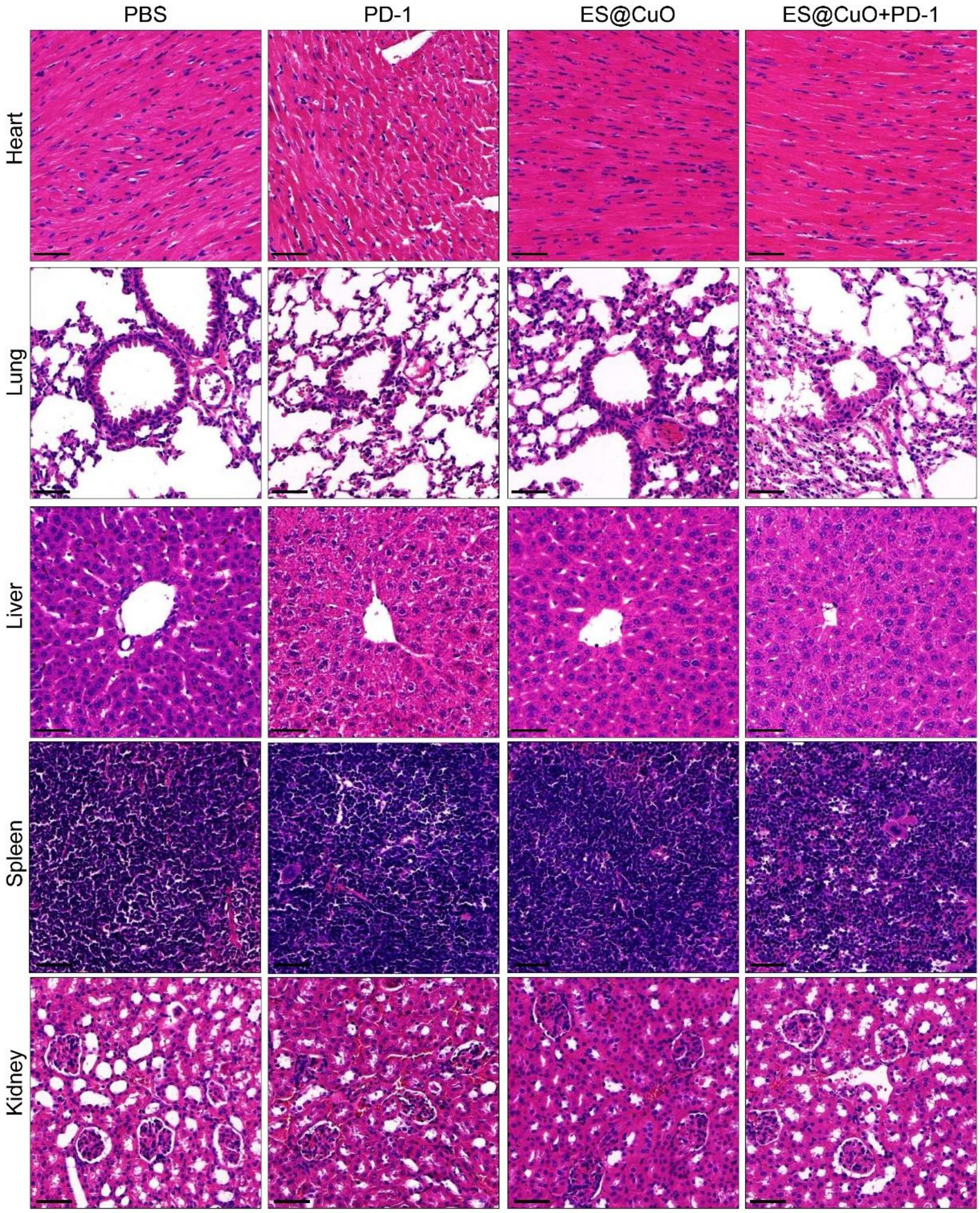

Fig. S22

Fig. S22. H&E staining of mice's heart, lung, liver, spleen, and kidney with different treatments. Scale bar: 50 μm.

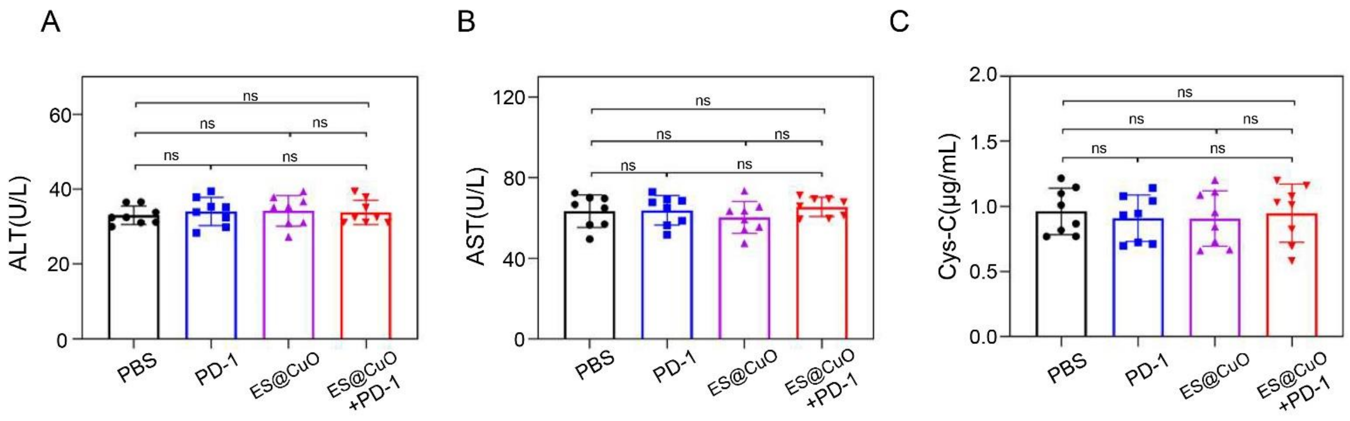

Fig. S23

Fig. S23. Biochemical analysis of the serum of mice with various treatments (n = 8). Data are shown as the mean ± SD; p values are calculated using an unpaired, 2-tailed student's t test with Welch's correction; ns, nonsignificant.

Fig. 3C

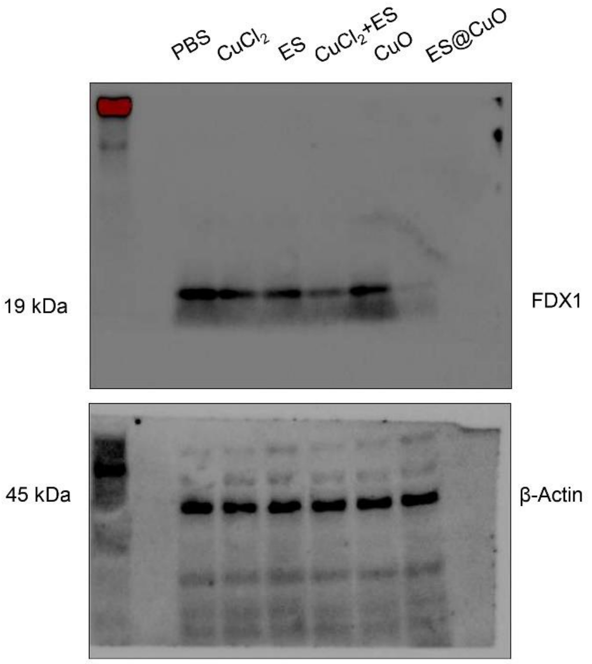

Fig. S12B

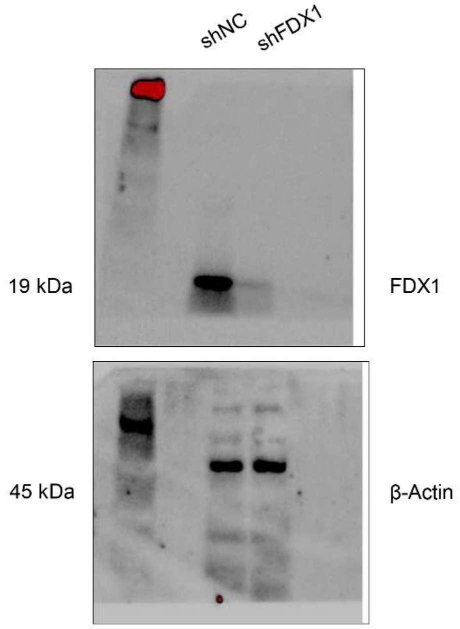

Fig. S24

Fig. S24. Uncropped western blot of figures.
